# Counteraction between Astrin-PP1 and Cyclin-B-CDK1 pathways protects chromosome-microtubule attachments independent of biorientation

**DOI:** 10.1038/s41467-021-27131-9

**Published:** 2021-12-01

**Authors:** Xinhong Song, Duccio Conti, Roshan L. Shrestha, Dominique Braun, Viji M. Draviam

**Affiliations:** 1 School of Biological and Chemical Sciences, Queen Mary, University of London, London, E1 4NS UK; 2 Department of Genetics, University of Cambridge, Cambridge, CB2 3EH UK; 3 Present Address: Department of Mechanistic Cell Biology, Max Planck Institute of Molecular Physiology, Otto-Hahn-Straße 11, 44227 Dortmund, Germany; 4 Present Address: Center for Cancer Research, National Cancer Institute, National Institutes of Health, Bethesda, MD 20892 USA

**Keywords:** Chromosome segregation, Kinetochores, Genomic instability, Mitotic spindle

## Abstract

Defects in chromosome-microtubule attachment can cause chromosomal instability (CIN), frequently associated with infertility and aggressive cancers. Chromosome-microtubule attachment is mediated by a large macromolecular structure, the kinetochore. Sister kinetochores of each chromosome are pulled by microtubules from opposing spindle-poles, a state called biorientation which prevents chromosome missegregation. Kinetochore-microtubule attachments that lack the opposing-pull are detached by Aurora-B/Ipl1. It is unclear how mono-oriented attachments that precede biorientation are spared despite the lack of opposing-pull. Using an RNAi-screen, we uncover a unique role for the Astrin-SKAP complex in protecting mono-oriented attachments. We provide evidence of domains in the microtubule-end associated protein that sense changes specific to end-on kinetochore-microtubule attachments and assemble an outer-kinetochore crescent to stabilise attachments. We find that Astrin-PP1 and Cyclin-B-CDK1 pathways counteract each other to preserve mono-oriented attachments. Thus, CIN prevention pathways are not only surveying attachment defects but also actively recognising and stabilising mature attachments independent of biorientation.

## Abstract (web summary)

Chromosome instability frequently occurs due to issues with chromosome-microtubule attachments. Here the authors show that the Astrin-PP1 and Cyclin-B-CDK1 pathways counteract each other to protect chromosome-microtubule attachments independent of biorientation.

## Introduction

Non-bioriented kinetochore-microtubule attachments predominate during early mitosis^1–3^ and are a step towards biorientation. Biorientation allows the pulling and segregation of chromosomes into two equal sets. Unresolved non-bioriented attachments such as syntelic (co-oriented), monotelic (mono-oriented) and merotelic (multi-oriented) attachments (Supplementary Fig. 1a) can cause chromosome missegregation, leading to nuclear atypia, transcriptional and cell cycle changes^4,5^. It is known that syntelic and merotelic attachments are resolved through multiple mechanisms: destabilisation of incorrect attachment (Aurora-B/Ipl1 mediated error correction pathway^6–9^), progressive restriction of attachment geometry (Dynein powered corona-stripping^10–14^) and microtubule-pulling or tension associated active stabilisation of attachments^15–17^. However, whether and how cells recognise and regulate mono-oriented kinetochore-microtubule attachments is not understood.

The significance of monotelic kinetochore geometry and orientation in ensuring the normal timing of biorientation and in preventing the formation of syntelic or merotelic attachment errors are evident in several in silico models^18–20^. Mono-oriented end-on attachments form in two ways: when kinetochores interacting with microtubule walls become tethered to microtubule ends (end-on conversion^21–23^), and when microtubules nucleate from kinetochores as pre-formed K-fibers^24,25^. Oscillations of mono-oriented chromosomes (including velocity, duration and amplitude) are similar to other chromosome oscillations^2,3,26^, but how mono-oriented attachments are protected without the opposing pull is unclear. One potential way to protect a mono-oriented kinetochore-microtubule attachment from Aurora-B mediated error correction could be by establishing tension through its sister kinetochore’s interaction with microtubule walls, as lateral kinetochore-microtubule interactions are immune to centromeric Aurora-B^27,28^. However, this is unlikely to be the primary mechanism for at least two reasons: the outer-kinetochore region of human cells maintains active Aurora-B kinase (pT232)^27,29^, which can destabilise lateral attachments^27^. Second, stable mono oriented kinetochores have been observed in a variety of conditions^23,25,26^. Other kinetochore-bound kinases, CDK1 and PLK1, have been implicated in stabilising chromosome-microtubule attachments^30^ but their impact specifically on mono-oriented attachments (independent of microtubule-mediated opposing pull) remains unclear. Polar ejection forces acting on chromosome arms are vital for the end-on conversion of mono-oriented kinetochore-microtubule attachments^31^, but the precise molecular players at the kinetochore that selectively maintains these end-on attachments are not known.

Pools of evolutionarily conserved phosphatases are delivered to the kinetochore^32,33^ by proteins capable of interacting with microtubules and essential for normal chromosome-microtubule attachment: CENP-E^34^, Astrin-SKAP/Kinastrin complex (referred as Astrin-SKAP here)^27,35^, SKA complex^36^ and KNL1/hSPC105/Blinkin (referred as KNL1 here)^37–39^. While KNL1/hSPC105/Blinkin is a constitutive kinetochore protein^37,38^, CENP-E is localised to kinetochores during prometaphase and metaphase but not anaphase^40^. On the other hand, the Astrin-SKAP/Kinastrin complex is recruited to end-on, but not lateral, kinetochores during prometaphase^23^ and is retained through metaphase and anaphase^41,42^, while the SKA complex steadily increases in levels at the kinetochore during prometaphase and is retained during anaphase^43–45^. Whether these phosphatase pools counteract distinct sets of kinases to control different stages of the chromosome-microtubule attachment process is not known.

Here we present evidence for how human cells exploit a localised kinase-phosphatase feedback loop to preserve mono-oriented end-on attachments, independent of biorientation. Through a targeted RNAi-screen, we uncovered an essential function of the Astrin-SKAP complex in preserving mono-oriented attachments even when the error-correction pathway is switched off. To understand how Astrin facilitates the sensing or stabilisation of mono-oriented attachments, we used time-lapse microscopy tools to assess Astrin arrival and departure at kinetochores. We report the role of CDK1 in regulating Astrin enrichment at end-on attachments without disrupting Aurora-B mediated error correction of attachments. To explain how the Astrin complex is specifically recruited to end-on attached kinetochores, we performed deletion studies and identified that the C-terminus of Astrin bears Aurora-B and CDK1 responsive protein domains that recognise outer-kinetochore changes without the need for Astrin binding to microtubules. We show that the Astrin-delivered pool of PP1 dephosphorylates a subset of CDK1 phosphorylation sites on Bub1, but not CENP-T or Ndc80/Hec1, revealing a mechanism linking the arrival of Astrin and departure of Mad1-Mad2 checkpoint proteins. In agreement, through Astrin mutants and colocalisation studies, we establish that Astrin-PP1 and Cyclin-B-CDK1 pathways form a negative feedback loop to maintain non-bioriented attachments, separate from the canonical Aurora-B mediated pathway for error correction.

## Results

### Astrin and SKAP protect mono-oriented KT-MT attachments

We find that blocking the recruitment of BubR1-B56 phosphatase, an enzyme crucial for end-on attachments in bipolar spindles^27,46^, disrupts the maintenance of mono-oriented end-on attachments (Supplementary Fig. 1b, c). This shows that phospho-signalling is likely to underpin the life of mono-oriented kinetochore-microtubule attachments. Hence, we set out to explore how Aurora-B mediated error-correction of syntelic and merotelic attachments coexist with the need to preserve monotelic attachments. We performed an RNAi-based screen of kinetochore proteins, particularly those delivering phosphatases, to identify candidates required to maintain non-bioriented end-on attachments in the absence of error-correction enzyme Aurora-B. For this purpose, we depleted kinetochore-microtubule bridging proteins using standardised siRNA oligos^27,47–49^ (Supplementary Table 1) and exposed cells to an Eg5 inhibitor (STLC or Monastrol) to induce monopolar spindles followed by brief exposure to ZM443479 and MG132 to inhibit Aurora-B and block anaphase onset, respectively. In control cells, this treatment results in monopolar spindles with kinetochores tethered to the tip of microtubule-ends forming a bouquet-like arrangement (Supplementary Fig. 1d). We assessed attachment status using three methods: (i) comparing kinetochore position at the end of microtubule (end-on interaction), (ii) monopolar bouquet-like arrangement of kinetochores and (iii) the presence or absence of Astrin crescents, a marker specific for stable end-on kinetochores independent of biorientation^27,35^. In this targeted screen, co-depletion of Ndc80/Hec1 and Nuf2 was used as a positive control as the Ndc80 complex is essential for all forms of kinetochore-microtubule attachments^50^. Depletion of chTOG1, Astrin-SKAP complex, SKA complex, EB1-EB3 complex and CLIP-170, all microtubule-associated proteins (MAPs), known to bind both the kinetochore and microtubule-ends (reviewed in Ref. ^30^), resulted in aberrant nuclei (multinucleated or misshapen) as expected, confirming chromosome missegregation induced by protein depletion (Supplementary Table 1). Our analysis of attachment status showed that chTOG1, EB1-EB3 complex and SKA complex are not essential for maintaining end-on attachments in the absence of Aurora-B activity (Supplementary Table 1 and Supplementary Fig. 1d), consistent with intact kinetochore-microtubule attachment in cells depleted of chTOG1^50,51^, EB1^47^, APC^47^ or SKA3^44,52^ in the absence of microtubule-destabilising cold treatment. Uniquely Astrin or SKAP depleted cells showed a reduction in the number of end-on attached kinetochores and loss of bouquet-like chromosome arrangement in monopolar spindles in this screen (Supplementary Fig. 1d; Supplementary Table 1). Thus, Astrin-SKAP, a kinetochore-associated MAP complex, is needed to maintain non-bioriented end-on attachments, independent of the error correction mechanism.

### CDK1 controls Astrin-mediated sensing of end-on attachments

**Fig. 1:**
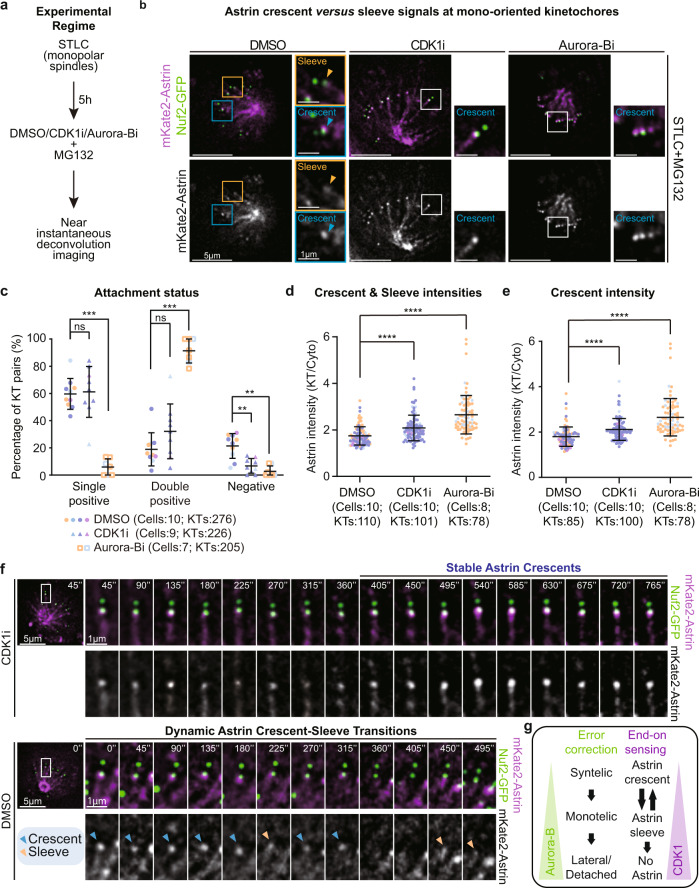
CDK1 activity controls Astrin-mediated sensing of end-on attachment. **a** Experimental regime shows near-instantaneous live-imaging of kinetochore fate soon after the addition of kinase inhibitors to understand end-on attachment sensing mechanisms in non-bioriented kinetochores. **b** Live cell images of monopolar spindles show monotelic kinetochores soon after DMSO or RO3666 treatment and syntelic kinetochores soon after ZM447439 treatment in cells co-expressing Nuf2-GFP and mKate2-Astrin. Cells were exposed to STLC for 5 h before adding CDK1 (RO3306) or Aurora-B (ZM447439) inhibitor or DMSO (control) with the proteasome inhibitor, MG132. Scale bar as indicated. Astrin crescent and sleeves are marked by blue and orange triangles, respectively. **c** Graph shows the percentage of monotelic (Astrin single-positive) or syntelic (Astrin double-positive) kinetochore pairs following inhibitor treatment as indicated using images as shown in **b**. **d, e**, Graph shows intensities of either Astrin crescents and sleeves (**d**) or Astrin crescents alone (**e**) in cells treated as in **a**. Colours in super-plot **c**–**e** represent independent experimental repeats. Black bars and whiskers in the graph **c**–**e** mark average value and standard deviation, respectively, across independent experimental repeats (*n* = 3 CDK1i and 2 Aurora-Bi repeats) independent. ‘*’ and ‘ns’ indicate statistically significant and insignificant differences, respectively, were determined using a Non-parametric two-sided Mann-Whitney test. The exact *p*-values can be found in Source data. **f** Time-lapse images of kinetochores show transitions in Astrin crescent *versus* sleeve at kinetochores of monopolar spindles in cells coexpressing mKate2-Astrin and Nuf2-GFP and treated with DMSO but not CDK1 inhibition. Cells were treated with STLC for 5 h and MG132 was added along with either CDK1 inhibitor or DMSO (control) as indicated and filming started soon after drug addition. The time interval between frames is 45 s. Astrin crescent and sleeves are marked by blue and orange triangles, respectively. *N* = 8 cells (DMSO) and 7 cells (R03306) from two independent repeats. Scale bar as indicated. **g** Illustration of separable roles for Aurora-B and CDK1 in regulating Astrin-mediated sensing of end-on attachments. Global reduction in CDK1 increases the amount of Astrin at kinetochores without disrupting error correction that is dependent on Aurora-B.

The heterotetrameric Astrin-SKAP complex enriches specifically at end-on (and not lateral) kinetochores^23^ and Astrin’s PP1 binding tail is important for maintaining stable end-on attachments^35^. We hypothesised that factors that control Astrin recruitment at non-bioriented kinetochores can reveal how cells sense and stabilise end-on attachments before biorientation. So, we searched for factors that control Astrin enrichment by studying the incidence and intensity of Astrin at kinetochores of monopolar spindles in fixed-cell studies (Supplementary Fig. 2a, b). As expected inhibiting the error-correction enzyme Aurora-B increased the percentage of Astrin double-positive kinetochore pairs showing an increase in syntelic attachments (Supplementary Fig. 2c). In addition, Aurora-B inhibition increased the amount of Astrin at kinetochores (Supplementary Fig. 2d), as reported^53^. Surprisingly, CDK1 inhibition increased the amount of Astrin at the outer-kinetochore (Supplementary Fig. 2d), without promoting syntelic attachments (Supplementary Fig. 2c). Thus CDK1 activity influences Astrin-mediated sensing of end-on attachments without disrupting error correction. To confirm these findings, we analysed live-cells coexpressing mKate2-Astrin and Nuf2-GFP (a kinetochore marker) soon after the addition of CDK1 inhibitor (Fig. 1a). In monopolar spindles of cells exposed to DMSO (solvent control), 80% of kinetochore pairs displayed mKate2-Astrin (Fig. 1b, c) with 60% as monotelic (single-positive) and 20% as syntelic (double-positive) kinetochore pairs (Fig. 1c). Consistent with fixed-cell studies (Supplementary Fig. 2), the percentage of syntelic attachments and the amount of kinetochore-bound Astrin were increased following Aurora-B inhibition (Fig. 1c, d). In contrast, CDK1 inhibition increased the amount of kinetochore-bound Astrin (Fig. 1d) but did not significantly increase the percentage of syntelic attachments (Fig. 1c), confirming CDK1’s role in controlling Astrin recruitment without disrupting error-correction. We have shown that Astrin localisation fluctuates between a crescent and sleeve shape at the outer-kinetochore of monopolar spindles^35^. As the change in outer-kinetochore signal of Astrin from ‘sleeve’ to ‘crescent’ is dynamic, we quantified changes in Astrin crescents alone and observed a significant increase in average Astrin intensities (crescent) following CDK1 inhibitor treatment (Fig. 1e). To verify the extent of reduction in Aurora-B or CDK1 activities following inhibitor treatments, we immunostained inhibitor-treated cells to examine the status of two kinetochore substrates: CENP-T pT47 (CDK1 substrate^54^) and Hec1 pS55 (Aurora-B substrate^55^). The levels of both CENP-T pS47 and Hec1 pS55 were significantly reduced following CDK1 and Aurora-B inhibitor treatments, showing effective inhibition of respective kinase activity within 10 min of exposure to inhibitors (Supplementary Fig. 2e–h). In conclusion, both fixed and live-cell studies show that, unlike Aurora-B, CDK1 activity regulates Astrin enrichment at end-on kinetochores without interfering with error correction mechanisms.

CDK1 is reported to be needed for establishing kinetochore-microtubule attachments^56^. So, we were surprised to observe an increase in the amount of kinetochore-bound Astrin following CDK1 inhibition, which suggests a role for CDK1 in blocking Astrin-mediated sensing of mono-oriented end-on attachments (Fig. 1d, e). Therefore, to investigate how CDK1 activity influences Astrin enrichment at kinetochores, we tracked mono-oriented kinetochores soon after the addition of CDK1 inhibitor using deconvolution time-lapse microscopy of monopolar spindles in cells coexpressing mKate2-Astrin and Nuf2-GFP. Careful temporal analysis of change in Astrin levels at mono-oriented end-on kinetochores showed dynamic changes in Astrin signals (crescent *versus* sleeve) in control (DMSO-treated) cells, as reported in unperturbed cells^35^ (Fig. 1f). However, in CDK1 inhibitor-treated cells, kinetochore crescents of Astrin did not transition into sleeves within 6 min of treatment (Fig. 1f). Thus, Astrin enrichment at mono-oriented end-on attachment is counteracted by CDK1 activity.

In summary, (i) Astrin-mediated dynamic sensing of nonbioriented end-on kinetochores is under the influence of CDK1, (ii) CDK1 inhibition induced Astrin enrichment does not impede error-correction and (iii) CDK1 and Aurora-B kinases influence the fate of kinetochore-microtubule attachments differently (Fig. 1g).

### Kinetochore-autonomous control of Astrin dynamics

**Fig. 2:**
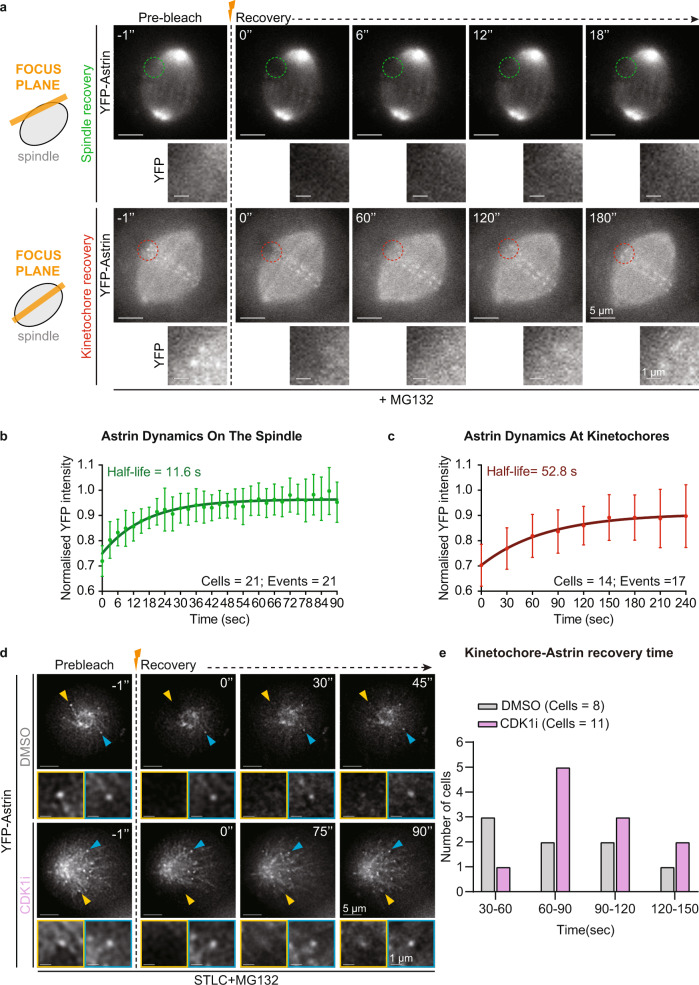
Kinetochore-autonomous regulation of Astrin dynamics at end-on attachments. **a** Representative time-lapse images of Fluorescence Recovery After Photobleaching (FRAP) of HeLa cells expressing YFP-Astrin following one hour of MG132 treatment prior to photobleaching. Cropped images show the recovery of YFP intensities after bleaching one spindle area (green circle) or one kinetochore (red circle). Cartoons on the left display a cross-sectional view of the spindle showing the plane of focus (orange bar) for microtubules (top) or kinetochores (middle) of the mitotic spindle (shown as a grey ellipse). Scale bar as indicated. **b** and **c**, Graphs show curves of normalised YFP fluorescence intensities on spindle (**b**) or kinetochores (**c**), respectively, versus time from FRAP experiments as in **a**. Lines and whiskers mark the average and standard deviation, respectively, from two independent experiments (kinetochore data) and three independent experiments (microtubules data). The calculated half-life time of recovery is indicated in each graph. **d** Representative time-lapse images of Fluorescence Recovery After Photobleaching (FRAP) of HeLa cells expressing YFP-Astrin, acquired once every 15 s, following one hour of STLC treatment prior to photobleaching in the presence of CDK1 inhibitor (CDK1i, RO3306) or DMSO (control) as indicated. Cropped images outlined in yellow and blue indicate bleached and unbleached (control) kinetochore, corresponding to yellow and blue arrowheads, respectively. Scale bar as indicated. **e** Kinetochore-bound Astrin recovery times with or without CDK1 inhibitor obtained using movies as in **d**. Recovery time was visually scored to indicate the first time point (30 s bin) where Astrin signal at the kinetochore was seen following the bleaching event. The values shown are from two independent experimental repeats.

Unlike mono-oriented end-on kinetochores where Astrin crescents and sleeves alternate dynamically, bioriented end-on kinetochores stably display Astrin crescents (Fig. 1 and Ref. ^35^). To explore whether Astrin-mediated sensing of end-on attachments continues to be dynamic at bioriented kinetochores, we measured Astrin turnover. Fluorescence Recovery After Photobleaching (FRAP) of YFP-Astrin showed that the recovery of YFP-Astrin signal intensities at kinetochore was significantly slower compared to spindle microtubules: 52.8 s (range of 29.5–252.4 s) at kinetochores vs. 11.6 s (range of 9.6–14.6 s) on microtubules (Fig. 2a–c). This indicates two distinct pools of Astrin: one at the outer kinetochore and the other along spindle microtubules. Importantly, the FRAP rates reveal a wide range in the half-lives of YFP-Astrin at kinetochores, compared to microtubules, indicating kinetochore-autonomous changes in Astrin crescent turnover.

The turnover rate of kinetochore-bound Astrin is at least 4-fold higher compared to turnover rates of GFP-tubulin at kinetochore bound microtubules (7–9 min^57–59^), suggesting that the dynamic outer kinetochore shell of Astrin is not directly related to microtubule plus-end assembly rates. Consistent with this idea, the turnover rate of YFP-Astrin on spindle microtubules is somewhat similar to the turnover rate of GFP-tubulin in non-kinetochore microtubules (21.5 s^57^). Thus, the distinct pool of Astrin at kinetochores and the rapid turnover of Astrin at kinetochores compared to the turnover of tubulin at kinetochore-microtubules show that Astrin forms a dynamic outer-kinetochore crescent when chromosomes are end-on attached.

We next explored the extent to which CDK1 can regulate the kinetochore-autonomous changes observed in Astrin crescent turnover. For this purpose, we extended the FRAP study to mono-oriented kinetochores in the presence and absence of the CDK1 inhibitor (Fig. 2d). As monopolar spindles are significantly more dynamic than bipolar spindles and are difficult to track due to spatial and temporal discontinuities in time-lapse movies^60^, we analysed the first time-point of signal recovery on photobleached kinetochores (Fig. 2d). Following DMSO treatment (control cells), YFP-Astrin signal recovery times were spread out between a range of 30–120 s. However, following CDK1 inhibitor treatment, the recovery times for Astrin crescent aggregated towards a mode value of 60–90 s (Fig. 2e). This shows that CDK1 inhibition does not abolish, but narrows the differences in Astrin dynamics across individual kinetochores, revealing the kinase’s specific role in regulating Astrin turnover at kinetochores. Together, these observations show that Astrin localisation at mono-oriented end-on kinetochores is dynamic, kinetochore-autonomous, and negatively regulated by CDK1 activity.

### CDK1 and Aurora-B differently control Astrin at the kinetochore

**Fig. 3:**
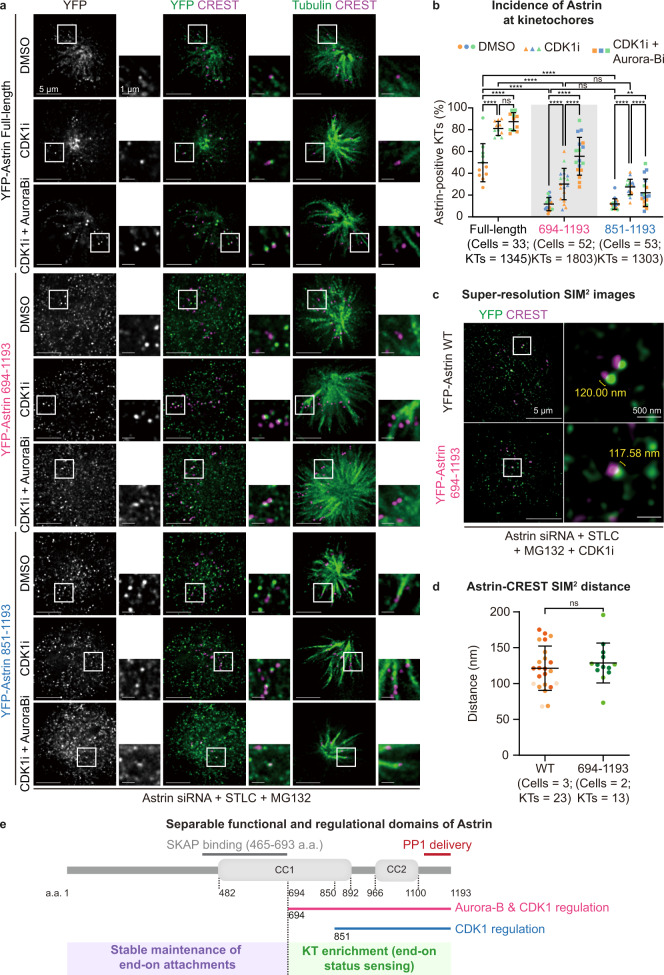
Molecular basis for Astrin’s dual role in maintaining and recognising mono-oriented end-on attachments. **a** Representative images of immunostained cells show the localisation of YFP tagged Astrin fragments (694–1193 or 851–1193, as indicated) following 5 h of STLC treatment to increase mono-oriented kinetochores and exposed to CDK1 inhibitor (RO3306) or Aurora-B inhibitor (ZM447439) for 15 min prior to fixation. Cells were immunostained with antibodies against GFP, Tubulin and CREST (as kinetochore marker). Only instances where YFP crescent signals are sandwiched between Tubulin and CREST signals are scored as positive for YFP-Astrin recruitment at the outer-kinetochore. White boxes mark the area of cropped images. Scale bar as indicated. **b** Graph shows the percentage of YFP-Astrin positive kinetochores in cells expressing YFP-Astrin WT or mutant and treated with CDK1i or Aurora-B inhibitors as indicated in **a**. **c** Representative super-resolution microscopy images of cells treated as in **a**. Measurements in crops indicate intra-kinetochore distances between peak intensities of CREST and YFP signals. **d** Graph shows the average intra-kinetochore distance between CREST and YFP signals in kinetochores of cells expressing Astrin full-length protein or a 694–1193 a.a fragment. Colours in super-plots **b** and **d** represent two (DMSO) or three (CDK1i or CDK1i and Aurora-Bi) independent experimental repeats (**b**) or cells (**d**), respectively. Horizontal black bars and whiskers mark average value and standard deviation, respectively, across two (WT) or three (fragment) independent experiments. ‘*’ and ‘ns’ indicate significant and insignificant statistical differences, respectively, as determined by the Non-parametric two-sided Mann-Whitney test. Exact *p*-values can be found in Source data. **e** Schematic of Astrin fragments used to dissect Astrin’s dual role in maintaining and sensing end-on attachments. Kinetochore binding C-terminus and microtubule-binding N- terminus of Astrin contribute to the sensing and maintenance of end-on attachments, respectively. The C-terminal region of Astrin that is responsible for sensing end-on kinetochores, bears at least two domains that are differently regulated by Aurora-B and CDK1 pathways.

How cells sense end-on attachments independent of biorientation is not clear, although it is known that the checkpoint proteins Mad2 and Mad1 leave the kinetochore soon after the formation of end-on attachment^22,23^, prior to biorientation^23^. As the localisation of the Astrin-SKAP complex is kinetochore-autonomous (Fig. 2), and the complex is enriched at end-on but not lateral kinetochores^27^, we hypothesised it as a strong candidate for directly recognising outer kinetochore changes specific to end-on attachments. So, we searched for the minimal mono-oriented kinetochore targeting domain in Astrin. Previous studies in bipolar spindles show the microtubule-binding and SKAP-interacting amino-terminus of Astrin as dispensable for Astrin’s localisation at bioriented kinetochores;^61,62^ whether this remains true at mono-oriented end-on kinetochores is not known. To address this, we expressed YFP tagged fragments of Astrin (694–1193 and 851–1193 a.a.) that lack the complex’s microtubule-binding region, and immunostained cells to study Astrin fragment localisation at mono-oriented kinetochores (Fig. 3a). In addition, we tested whether the localisations of these Astrin fragments are sensitive to either CDK1 or Aurora-B activity, similar to full-length Astrin protein, to determine how Astrin localisation was regulated by the two kinases. Immunostaining revealed that although both the C-terminal fragments of Astrin can localise at mono-oriented kinetochores (sandwiched between tubulin and CREST signals, Fig. 3a), their incidence at end-on kinetochores (YFP-Astrin fragment positive kinetochores) was significantly different and also significantly reduced compared to the full-length control (Fig. 3b). Similar to full-length Astrin, the incidence of the two fragments at mono-oriented kinetochores increased significantly following CDK1 inhibition (Fig. 3b). However, only the 694–1193 a.a fragment (but not the 851–1193 a.a fragment) showed a further increase in its incidence at kinetochore following Aurora-B coinhibition, showcasing the role of Astrin’s 694–851 a.a. region in responding to Aurora-B kinase activity (Fig. 3e, cartoon). These studies provide molecular evidence for separate CDK1 and Aurora-B mediated control of Astrin recruitment at kinetochores.

We next assessed the stability of mono-oriented end-on attachments. Co-immunostaining for Astrin and tubulin showed a significant reduction in the percentage of end-on attachments in cells expressing the Astrin fragments compared to those expressing the full-length protein (Supplementary Fig. 3a), in agreement with the reduced incidence of YFP-Astrin C-terminal fragments at mono-oriented kinetochores (Fig. 3b). Moreover, both the C-terminal Astrin fragments were recruited only to a subset of end-on kinetochores (as ascertained using CREST and tubulin costaining), and this subset of Astrin fragment positive end-on kinetochores could be significantly increased by inhibiting CDK1 (Supplementary Fig. 3b). These immunostaining studies show that both the C-terminal fragments of Astrin can be recruited, but not stably present at mono-oriented end-on kinetochores in the presence of CDK1 activity. These observations reveal an important role of Astrin’s N-terminus in the maintenance of mono-oriented end-on attachments, and Astrin’s C-terminus in the enrichment of Astrin-SKAP at kinetochores in a CDK1 dependent manner.

To assess whether the C-terminal 694–1193 a.a fragment and full-length Astrin protein recruitment are associated with recognising similar changes at the outer-kinetochore we turned to super-resolution microscopy. Using Lattice SIM^2^ (with a lateral super-resolution of 60 nm), we measured intra-kinetochore distances between the N-terminus of Astrin and inner kinetochore/centromeric boundary (CENP-C). Cells expressing YFP-Astrin full-length or the 694–1193 a.a. fragment were co-immunostained using anti-GFP and anti-Tubulin antibodies and CREST anti-sera that recognises CENP-A, CENP-B and CENP-C^63^ (Fig. 3c). Lateral distance measurements along the kinetochore-centromere axis showed similar positioning of the full length and 694–1193 a.a. fragment of Astrin at the outer kinetochore, on an average 120–125 nm away from the CREST anti-sera epitope site (Fig. 3d).

In summary, unlike bioriented kinetochores, mono-oriented end-on kinetochores require the microtubule-binding N-terminus of Astrin for Astrin’s stable localisation at kinetochores. Although the end-on kinetochore binding/sensing region of Astrin resides in the C-terminus of Astrin, the microtubule-binding N-terminus is required for the maintenance of end-on attachments which in turn influences Astrin’s stable enrichment at kinetochores. In alignment, CDK1 inhibition can increase the incidence of Astrin C-terminus at kinetochore. We propose the C and N- termini of Astrin contribute to the sensing/recognition and maintenance of end-on attachments, respectively (Fig. 3e). The C-terminal region of Astrin that is responsible for recruiting Astrin at end-on kinetochores, bears at least two physically separable domains that are differently regulated by Aurora-B and CDK1 pathways (Fig. 3e).

### A 273 a.a region of Astrin can sense end-on attachments

**Fig. 4:**
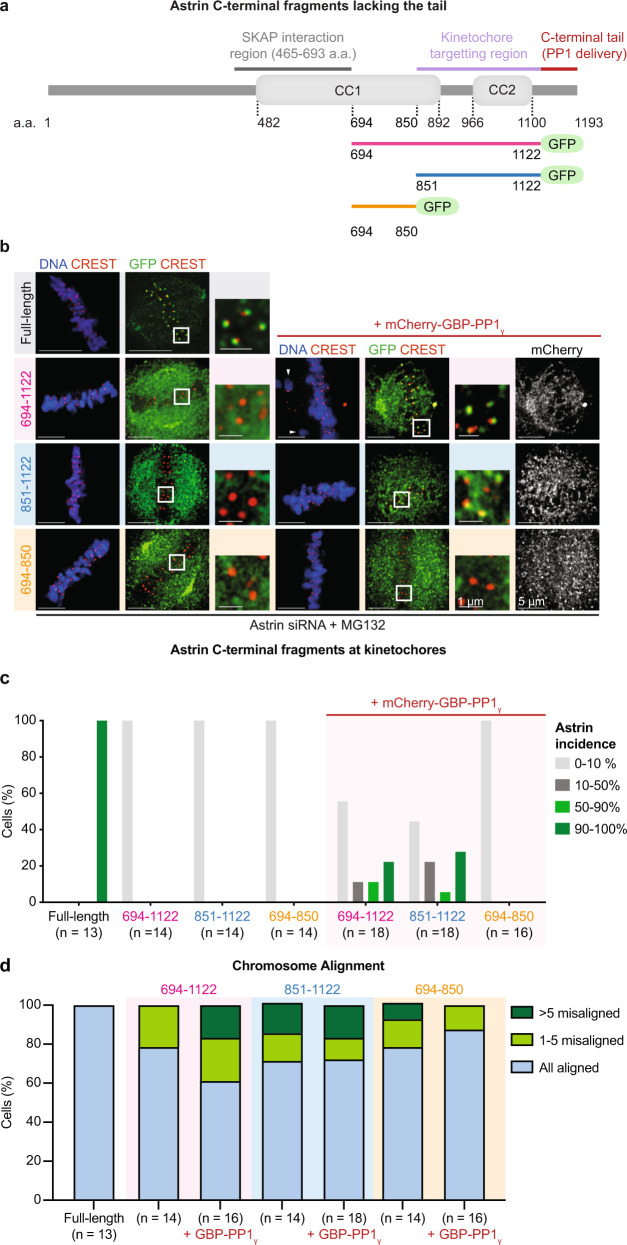
A 273 a.a region of Astrin is sufficient to sense outer-kinetochore changes at end-on attachments. **a** Schematic of Astrin fragments was used to ascertain minimal kinetochore targeting domain of Astrin. **b** Representative images of immunostained cells show the localisation of GFP tagged Astrin fragments (as indicated) in the presence or absence of mCherry-GBP-PP1 following an hour of MG132 treatment prior to fixation. Cells were immunostained with antibodies against GFP, mCherry and CREST (a kinetochore marker) and co-stained with DAPI. White boxes mark the area of cropped images. Scale bar as indicated. White arrowheads mark unaligned chromosomes quantified in **d**. **c**, Bar graphs show the percentage of cells displaying GFP-tagged fragments of Astrin, as indicated, in the form of outer-kinetochore crescents at 90–100%, 50–90%, 10–50% or 0–10% of aligned kinetochores in images as in **b**. Cells coexpressing mCherry-GBP-PP1 are marked separately. **d**, Bar graph shows the percentage of cells with chromosome alignment defects in cells expressing GFP-C-terminal tagged fragments of Astrin with or without mCherry-GBP-PP1 and treated as in **b**. To assess the extent of misalignment, cells were segregated into three bins: all chromosomes aligned, 1–5 misaligned chromosomes and >5 misaligned chromosomes. Values in **c** and **d** are from three independent experimental repeats.

As metaphase kinetochores stably recruit the C-terminal region of Astrin^61^, we used bipolar spindles to extend our deletion mutant studies for further narrowing down the minimal region of Astrin that can be recruited to kinetochores. In this study, we deleted the C-terminal tail of Astrin, as we have shown that the tail is needed to deliver a pool of PP1 and not for kinetochore localisation per se^35^. So, we exploited GBP-PP1 (GFP-Binding Protein fused to PP1) to constitutively deliver a pool of PP1 near Astrin-GFP’s C-termini in the absence of Astrin’s C-terminal tail (Fig. 4a)^35^. Using the tail deletion approach, we first asked whether the N-terminus of Astrin (1–693 a.a.) that forms the Astrin-SKAP tetramer and binds to microtubule walls^62^ is essential for the complex’s kinetochore localisation. Neither the Astrin-SKAP interaction domain nor Astrin’s microtubule-binding domain is needed to target Astrin at metaphase kinetochores, as 694–1122 a.a of Astrin-GFP can be recruited to kinetochores when coexpressed with mCherry-GBP-PP1 (Fig. 4b). Through further deletions, we narrowed down a 273 a.a stretch (851–1122 a.a) of Astrin as the shortest kinetochore targeting region. Interestingly, both the 694–1122 and 851–1122 a.a fragments of Astrin, coexpressed with mCherry-GBP-PP1, showed a reduction in the incidence and intensities of Astrin crescents compared to Astrin full-length protein (Fig. 4c and Supplementary Fig. 4a, b).

These findings show that although 851–1122 a.a. of Astrin is sufficient to recognise outer kinetochore changes specific to end-on kinetochores, the microtubule-binding N-terminus of Astrin is important for forming the complete Astrin crescent at the outer kinetochore.

Metaphase cells expressing Astrin-GFP fragments (694–1122 or 851–1122 a.a.) showed several unaligned kinetochores, highlighting the need for the full outer-kinetochore shell of Astrin to ensure the stable maintenance of congress chromosomes (Fig. 4d), consistent with our findings in mono-oriented kinetochores (Fig. 3). In summary, the microtubule-binding domain of Astrin is dispensable for Astrin’s recruitment to kinetochores, although this domain works closely with the C-terminal kinetochore-targeting region to build Astrin crescents specifically at end-on kinetochores.

### Astrin-PP1 lowers Bub1 phosphorylation linked to Mad1 level

To understand how mono-oriented attachments are protected from the premature action of error-correction pathways, we searched for downstream substrates of the Astrin delivered PP1 pathway at kinetochores. A signal transduction cascade including MPS1-(KNL1-MELpT)-Bub1-Mad proteins, operates closely attenuating checkpoint signals induced by MPS1 (reviewed in^64^). While we know how this checkpoint silencing cascade responds to the departure of checkpoint kinases^64^, we do not know how this cascade responds to the arrival of Astrin-PP1 phosphatase at end-on kinetochores.

**Fig. 5:**
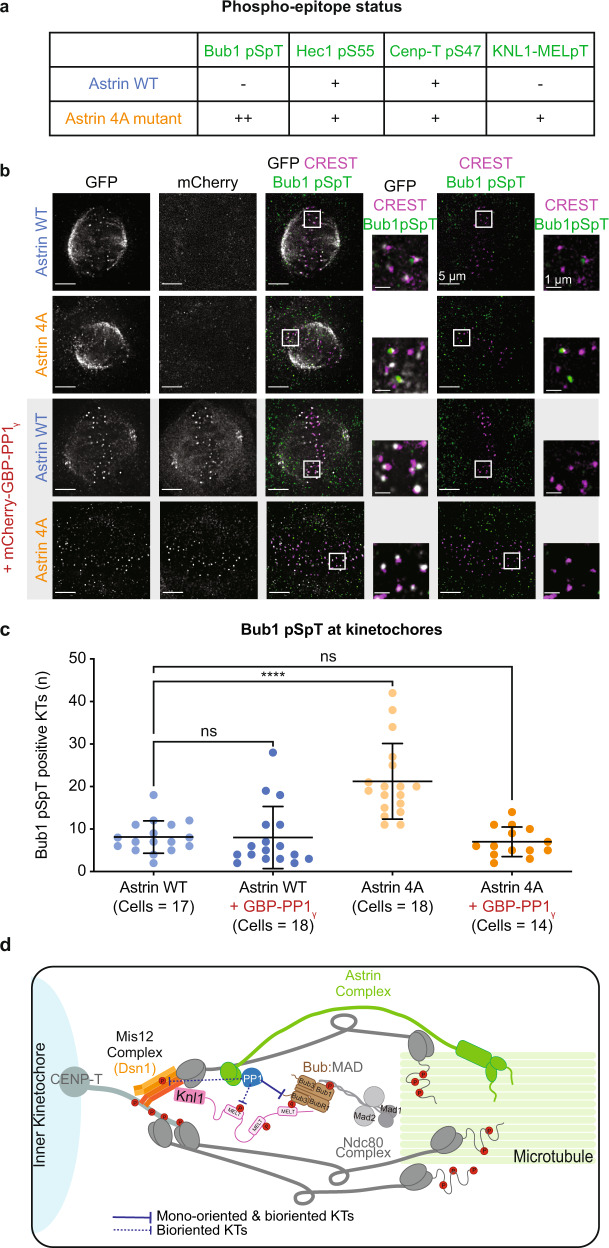
Astrin delivered PP1 lowers Bub1 phosphorylation linked to checkpoint silencing in mono-oriented and bi-oriented kinetochores. **a** Table summarises the phosphorylation status of various phospho-epitopes at the outer kinetochore. ‘+’ or ‘−’ refers to phospho-epitope observed in more or less than 20% of kinetochores, respectively, as shown in Supplementary Fig. 5. **b** Images show the extent of phosphorylated Bub1 (pSpT) at kinetochores of cells depleted of endogenous Astrin and expressing either Astrin WT-GFP or Astrin 4A-GFP mutant alone or along with mCherry-GBP-PP1 as indicated. Cells were treated with MG132 for an hour prior to fixation for immunostaining with antibodies against GFP, Bub1 pSpT phospho-epitope and CREST anti-sera (a centromere marker). Scale bar as indicated. **c** Graph showing the number of kinetochores positive for phosphorylated Bub1 pSpT, determined using whole spindle Z-stacks of images as shown in **b**. Each dot represents values from one cell. Bars and whiskers represent mean and standard deviation, respectively, across cells from three independent experimental repeats. “*” and “ns” indicate significant and insignificant statistical differences, respectively, as determined using a Kruskall-Wallis test with Dunn’s multiple comparisons. Mean rank differences are available in the Source data. **d** Model of dephosphorylation events that depend on the recruitment of Astrin-PP1 at end-on kinetochores. Astrin-PP1 alters the phosphorylation of (**i**) Bub1 (brown) responsible for positioning Mad1 near KNL1-MELpT, (**ii**) Dsn1 of Mis12 complex (orange), and (**iii**) KNL1-MELpT (pink) sites but not those of Ndc80 (S55) or CENP-T (S47) sites (grey), indicating a spatially restricted dephosphorylation of substrates. When PP1 delivery by Astrin is compromised, Bub1 dephosphorylation is impaired on both mono-oriented and bioriented kinetochores, while KNL1 dephosphorylation is only impaired on bioriented kinetochores.

The feedback loop involved in rapidly silencing the checkpoint is likely to be closely tied to end-on attachment sensing mechanisms, as Mad2 and Mad1 checkpoint proteins are known to rapidly leave end-on kinetochores before biorientation^22,23^. So, we took advantage of Astrin 4A mutant with a crippled PP1-docking site^35^ as a molecular probe to disrupt Astrin-mediated PP1 delivery and study its impact on phospho-events at the outer-kinetochore. Immunostaining for phospho-epitopes showed that cells expressing YFP-Astrin WT or YFP-Astrin 4A mutant display no significant changes in the incidence or intensity of Hec1 pS55 (Aurora-B substrate)^65^ or CENP-T pS47 (CDK1 substrate)^54^ at kinetochores (Fig. 5a and Supplementary Fig. 5a, b and g, h). However, compared to YFP-Astrin WT expressing cells, YFP-Astrin 4A mutant expressing cells display a noticeable increase in KNL1(MELpT)^66,67^ and Bub1(pSpT)^68^ positive kinetochores (Supplementary Fig. 5c–f and Fig. 5a). Immunostaining for KNL1 or Hec1 showed no obvious differences in their kinetochore localisation in cells expressing either Astrin-WT or 4A mutant (KNL1: WT: *n* = 10 cells; 4A: *n* = 10 cells; Hec1: WT: *n* = 10 cells; 4A: *n* = 10 cells; Supplementary Fig. 6a, b). The proportion of Bub1 positive kinetochores or Bub1 intensities were also not significantly different between cells expressing YFP-Astrin WT or 4A mutant (Supplementary Fig. 6c–e)

The specific increase in the proportion of Bub1 (pSpT) and KNL1(MELpT) positive kinetochores in YFP-Astrin 4A mutant expressing cells (Supplementary Fig. 5d, f) is in agreement with the increase in Mad2 and Zw10 displaying metaphase kinetochores following YFP-Astrin 4A mutant expression^35^. So, we tested whether the increase in the levels of phosphorylated Bub1 (pSpT; CDK1 substrate^68^) in Astrin-GFP 4A mutant expressing cells could be rescued by co-expressing mCherry-GBP-PP1. Immunostaining studies show a reduction in Bub1(pSpT) positive kinetochores in cells coexpressing Astrin-GFP 4A and GBP-PP1 compared to those expressing Astrin-GFP 4A alone (Fig. 5b, c), confirming a rescue of Astrin-4A mutant phenotype.

We investigated mono-oriented kinetochores for changes in Bub1 or KNL1 phosphorylation following Astrin 4A mutant expression to assess whether mono-oriented kinetochores followed the same trend as bioriented kinetochores. Immunostaining of cells depleted of endogenous Astrin and expressing either YFP-Astrin WT of 4A mutant, treated with STLC, showed that the incidence of Bub1(pSpT), but not KNL1(MELpT), signals are increased on mono-oriented kinetochores of cells expressing Astrin-4A mutant compared with Astrin WT (Supplementary Fig. 7). KNL1(MELpT) levels remained high in the vast majority of kinetochores of monopolar spindles in cells expressing either YFP-Astrin WT or 4A mutant (Supplementary Fig. 7c, d). This shows that a change in Bub1(pSpT) levels following YFP-Astrin 4A mutant expression is common to both mono-oriented and bioriented kinetochores, whereas the change in KNL1(MELpT) is specific to bioriented kinetochores.

Thus, a pool of PP1 phosphatase delivered by Astrin can promote the dephosphorylation of a subset of outer-kinetochore substrates and control the fate of mono-oriented and bioriented kinetochores by working closely with the KNL1-Bub1-MAD1 signalling cascade (Fig. 5d).

### CDK1 and Astrin-PP1 counteract at the outer-kinetochore

**Fig. 6:**
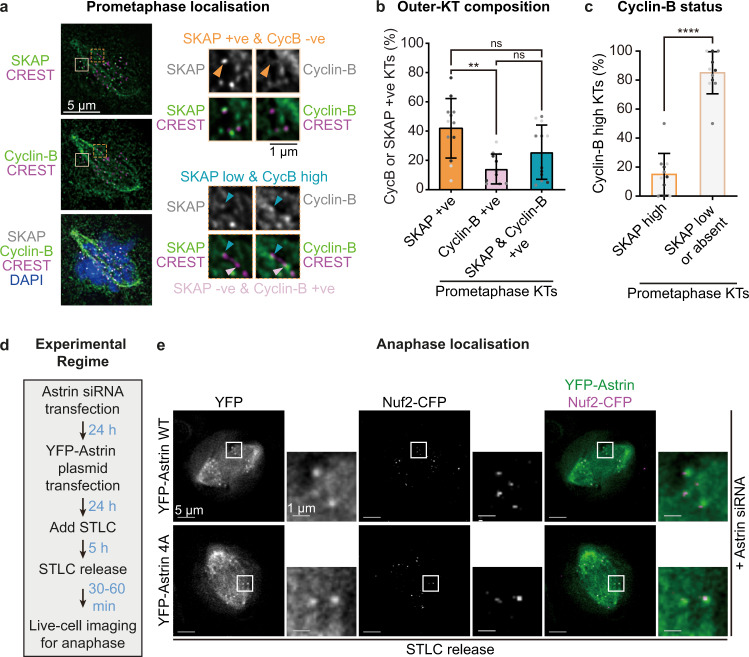
CDK1 and Astrin-PP1 counteract each other at the outer-kinetochore. **a** Images of RPE1 cells immunostained with antibodies against Cyclin-B and SKAP, and CREST anti-sera, and co-stained with DAPI for DNA. Arrowheads mark kinetochores with low SKAP and high Cyclin-B intensities (blue), SKAP-positive (+ve) and Cyclin-B-negative (−ve) kinetochore (orange), SKAP-negative (-ve) and Cyclin-B positive (+ve) kinetochore (pink). Scale bar as indicated. **b** Graph of the percentage of kinetochores enriched for either Cyclin-B or SKAP or both, ascertained using images as in **a**. ‘*’ and ‘ns’ indicates significant and insignificant statistical difference, respectively, determined using Non-parametric Kruskal-Wallis H test combined with Dunn’s multiple comparisons test. **c** Graph of percentage of Cyclin-B positive kinetochores with SKAP-high or −low intensities as in **a**. ‘*’ indicates statistical difference tested with two-tailed Mann Whitney test. Height of bars and whiskers (**b** and **c**) mark average value and standard deviation, respectively. Dots in the same shade of grey are from the same experimental repeat (*n* = 723 kinetochores, 12 cells and 3 independent experimental repeats). **d** Experimental regime to assess anaphase kinetochores: Astrin siRNA transfected cells coexpressing Nuf2-CFP (kinetochore marker) and either YFP-Astrin WT or YFP-Astrin 4A mutant were exposed to STLC for 5 h to synchronise monopolar spindles. After STLC was washed out to release cells into anaphase for live-cell imaging. **e** Representative anaphase live-cell images of mono-oriented kinetochores displaying YFP-Astrin WT or 4A crescents in cells treated as in **d** Sample size: WT (4 cells) and 4A (7 cells) from 2 independent experimental repeats. Scale as indicated.

The dephosphorylation of Bub1 by Astrin-PP1 (Fig. 5 and Supplementary Fig. 7) and the enrichment of Astrin following CDK1 inhibition (Fig. 1) indicate an opposing relationship between the activities of CDK1 kinase and Astrin delivered pool of PP1 phosphatase. To test this hypothesis, we first probed^35^ whether the arrival of Astrin-SKAP and departure of Cyclin-B-CDK1 at kinetochores are correlated. Immunostaining studies of RPE1 cells using antibodies against SKAP and Cyclin-B revealed prometaphase kinetochores displaying either SKAP or Cyclin-B signals (Fig. 6a, b), suggesting a departure of Cyclin-B in Astrin-SKAP enriched kinetochores. This was confirmed by quantification of Cyclin-B status at kinetochores enriched for SKAP: Kinetochores displaying high levels of Cyclin-B rarely displayed high levels of SKAP, instead, they frequently displayed low or no SKAP at kinetochores (Fig. 6c), demonstrating that Cyclin-B is significantly reduced at kinetochores enriched for the Astrin-SKAP complex.

To probe whether Astrin-PP1 and Cyclin-B-CDK1 pathways constitute a negative feedback loop, we studied Astrin-4A localisation in anaphase when CDK1 activity is reduced through Cyclin-B degradation^69^. To address this, we set up the synchronised release of cells into anaphase through the STLC arrest and release regimen to image segregating anaphase kinetochores^35^ (Fig. 6d). Anaphase cells coexpressing Nuf2-CFP and YFP-Astrin 4A showed normal kinetochore localisation of the Astrin-4A mutant at mono-oriented separated sister kinetochores (Fig. 6e). Comparing the proportion of anaphase kinetochores displaying Astrin-WT or 4A mutant, using live-cell imaging, showed no significant difference between Astrin-WT and 4A mutant recruitment (WT: *n* = 3 cells and 40 KTs; 4A: *n* = 7 cells and 70 KTs). This live-cell study shows that during anaphase, when cells experience very low to no CDK1 activity, Astrin-mediated delivery of PP1 is dispensable for Astrin’s enrichment at end-on kinetochores.

**Fig. 7:**
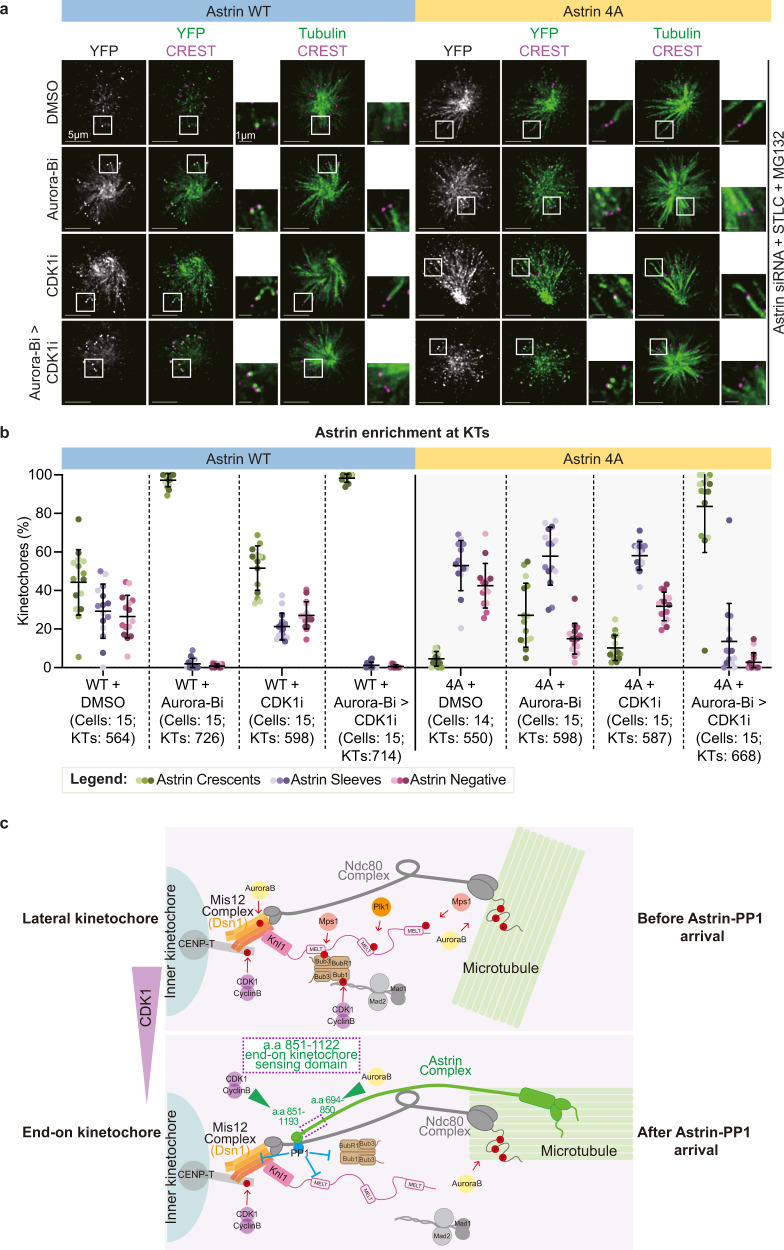
Aurora-B and CDK1 pathways separately counteract Astrin at the outer kinetochore. **a** Representative images show YFP-Astrin 4A mutant localisation at kinetochores of monopolar spindles sequentially exposed to Aurora-B and CDK1 inhibitors. Cells depleted of endogenous Astrin and expressing YFP-Astrin WT or 4A mutant as indicated were treated with STLC for 45 min followed by MG132 and DMSO or ZM447439 (Aurora-Bi) or Roscovitine (CDK1i) or sequential addition of Roscovitine into ZM447439 (ZM447439 alone for 15 min with a Roscovitine supplementation for an additional 15 min, Aurora-Bi > CDK1i). Cells were immunostained with antibodies against GFP and Tubulin and CREST anti-sera. Scale bar as indicated. **b** Scatter plot of Astrin recruitment status showing the proportion of Astrin Crescent or Sleeve or no Astrin signal (Astrin Negative) bearing kinetochores in cells expressing YFP-Astrin WT or 4A mutant as indicated and treated with inhibitors as in **a**. Black bars and whiskers mark average value and standard deviation, respectively, across experimental repeats (*n* = 4 repeats). Each dot represents a value from one cell. The colours of dots in **b** represent experimental repeats. **c** Cartoon of Astrin regulation at end-on kinetochores: Astrin deliver a pool of PP1 that counteracts a subset of spatially-limited phosphorylation events by multiple kinases (CDK1, Aurora-B or PLK1). CDK1 and Aurora-B pathways (green arrowheads) regulate the kinetochore binding of two separable regions at Astrin C-terminus. Inhibiting CDK1 and Aurora-B activities supersedes the need for Astrin-mediated PP1 delivery, revealing a feedback loop that protects non-bioriented end-on attachments from kinase activities.

To directly test whether one of the main roles of Astrin delivered PP1 is to counteract CDK1 substrate phosphorylation at the kinetochore and thereby, retain Astrin at mono-oriented kinetochores, we probed whether CDK1 inhibition will allow the enrichment of Astrin 4A mutant at mono-oriented end-on kinetochores^35^. Immunostaining studies showed that Astrin 4A mutant can be maintained at non-bioriented end-on kinetochores following the sequential inhibition of CDK1 after Aurora-B inhibition (Fig. 7a). Consistent with deletion mutant studies (Fig. 3), inhibition of both the Aurora-B and CDK1 pathways resulted in the normal recruitment of Astrin 4A mutant, as assessed by the percentage of Astrin crescent and sleeve structures at mono-oriented kinetochores (Fig. 7b). A significant increase in the incidence of Astrin 4A mutant crescents was observed following the co-inhibition of CDK1 and Aurora-B, compared to Aurora-B alone (Fig. 7b). We conclude that a key role of the Astrin-delivered pool of PP1 is to counteract the Cyclin-B-CDK1 pathway at the outer-kinetochore (Fig. 7c), which in turn promotes Astrin’s enrichment and stabilises non-bioriented kinetochore-microtubule attachments.

## Discussion

Here, we report that non-bioriented end-on attachments are sensed and protected, independent of biorientation mechanisms. We show that Astrin-mediated sensing of end-on attachment is dynamic. We identify a short 273 a.a. region of Astrin as sufficient to recognise outer-kinetochore changes specific to mature end-on attachments. Of the kinetochore associated MAPs known to deliver PP1, Astrin’s role is unique: Astrin-PP1 is recruited specifically at end-on attached kinetochores (Fig. 7c), unlike CENP-E motor that binds to PP1 and delivers CLASP to naked unattached kinetochores^70^. Although the microtubule-associated SKA complex can enhance Ndc80-microtubule coupling and stability of bioriented attachments^71^, similar to the Astrin-SKAP complex^35^, neither the SKA complex nor CENP-E is important for maintaining end-on attachment in the absence of Aurora-B (this study). Taken together, Astrin’s microtubule-binding domain (through its N-terminus), its PP1 delivery role (through its C-terminal tail) and outer-kinetochore changes sensing role (through its C-terminal coiled-coil region) together bring a set of unique kinetochore-microtubule bridging function (Fig. 7c), showing how Astrin-SKAP domains recognise and stabilise end-on kinetochore-microtubule attachments independent of biorientation.

We find that CDK1 activity controls Astrin enrichment at non-bioriented kinetochores, while Astrin-delivered PP1 controls a subset of CDK1 phospho-sites at the kinetochore. Moreover, the lack of Astrin-delivered pool of PP1 can be overcome by counteracting CDK1. These three lines of evidence show the entangling of CDK1 kinase and Astrin-PP1 phosphatase pathways, in a feedback loop, appropriate for rapid recognition and stabilisation of end-on attachments, without interfering with biorientation mechanisms. This is consistent with a role for CDK1 in establishing attachments (reviewed in^30^) and Astrin-SKAP complex’s role in maintaining, but not forming, end-on attachments^23^.

How does CDK1 inhibition regulate Astrin-SKAP localisation? CDK1 activity can strip the fibrous corona from outer kinetochore^12^, impair the centromeric localisation of Chromosome Passenger Complex (including Aurora-B)^72^ and reduce the level of PLK1 at kinetochores^73,74^. Of these three mechanisms, our work so far supports a close link between the physical stripping of fibrous corona and the enrichment of Astrin as the corona proteins ZW10 and Mad2 are incompletely removed from the kinetochores of Astrin-4A expressing cells^35^. The other two mechanisms may not have a direct role for the following reasons: inhibiting Aurora-B alone fails to rescue Astrin 4A mutant’s localisation at the kinetochore (this study and^35^), suggesting that Aurora-B is unlikely to be the sole downstream component of CDK1 in counteracting Astrin delivered pool of PP1. Similarly, inhibiting PLK1 activity destabilises chromosome-microtubule attachments^75,76^, which would deplete and not enrich Astrin. Moreover, the timing of corona stripping and the release of Cyclin-B-CDK1 from kinetochores are events that are temporally coincident with Astrin’s arrival at kinetochores and they occur during prometaphase independent of inter-kinetochore stretching. In contrast, PLK1 is retained at stretched kinetochores during metaphase^77,78^ when Astrin-PP1 interaction is maximally achieved^35^. Thus, CDK1 mediated regulation of Astrin-SKAP recruitment represents a novel end-on attachment recognition and stabilisation pathway that is coincident with the stripping of the fibrous corona, another event that precedes biorientation.

Three lines of evidence shed light on how Astrin recruitment is restricted to end-on attached kinetochores. First, Astrin fragment (694–1193 and 851–1193 a.a) studies show that the impact of Aurora-B and CDK1 activities on Astrin are separable, confirming an Aurora-B independent role for CDK1 in regulating Astrin localisation at mono-oriented end-on attachments. Second, inhibition of CDK1, in addition to Aurora-B is required for the kinetochore enrichment of Astrin 4A mutant with a crippled PP1 docking motif. Although the precise molecular reason for why a reduction in CDK1 activity induces an increase in Astrin 4A levels at the kinetochore is not yet known, Astrin-delivered PP1 counteracts a subset of CDK1-mediated phosphorylation changes at the kinetochore. Collectively, they support our model that Astrin-delivered PP1 counteracts CDK1-mediated changes at the outer kinetochore which in turn promotes Astrin recruitment and stabilisation of mono-oriented end-on attachments (Fig. 7c).

The close association between the mechanism to sense non-bioriented end-on attachments and the signalling cascade that silences the mitotic checkpoint, through Astrin-PP1, may also be relevant for checkpoint silencing in meiosis I where homologs are separated while sister kinetochores remain mono-oriented and end-on tethered following end-on conversion^79,80^. Uncovering spatially localised mechanisms for controlling kinetochore-microtubule attachment stability, without invoking whole-cell changes, is important as chromosomal instability can be restored by tweaking chromosome-microtubule attachment stability that’s lost in most aneuploid cancer cells^59^.

## Methods

### Cell culture and drug treatments

HeLa cells (ATCC) cultured in Dulbecco’s Modified Eagle’s Media and RPE1 cells (ATCC) cultured in Ham’s-F12 were supplemented with 10% FCS and antibiotics (Penicillin and Streptomycin). HeLa FRT/TO Venus-BubR1 (WT and mutant) were induced with Doxycyline 24 h before fixation. All transfection studies were performed using HeLa cells, except for the Cyclin-B and SKAP co-immunostaining, which was performed using RPE1 cells. For live-cell imaging studies, cells were seeded onto 4-well cover glass chambered dishes (Lab-tek; 1064716) or µ-Slide 8 Well ibiTreat dishes (Thistle Scientific; 80826). HeLa and RPE1 cell lines were tested and confirmed free of Mycoplasma^81^. For inhibition studies, cells were treated with 10 µM RO3306 (1305, TOCRIS), 20 µM STLC (83265,TOCRIS), 10 µM ZM447439 (2458, TOCRIS), 100 nM Taxol (T7191, SIGMA-ALDRICH), 10 µM Monastrol (CAS 254753-54-3, TOCRIS) or 10 µM MG132 (1748, TOCRIS). For monopolar spindle studies, STLC or Monastrol treatment was for 45 min to 5 h and then supplemented with MG132 and kinase inhibitors for 15′ to 30′ prior to fixation. For bipolar spindle studies, MG132 treatment was for 1 h.

### Plasmid and siRNA Transfection

siRNA transfection was performed using Oligofectamine (Fisher; 12252011) according to the manufacturer’s instructions. Astrin siRNA oligo (UCCCGACAACUCACAGAGAAAUU) and Negative control siRNA (12,935–300) were from Invitrogen. Plasmid transfection was performed using TurboFect (Fisher; R0531) or DharmaFECT duo (Dharmacon; T-2010) according to manufacturer’s instructions. In addition to the standard protocol, after 4 h of incubation, the transfection medium was removed and a fresh selected pre-warmed medium was added to each well. In co-transfection studies, eukaryotic expression vectors encoding Astrin-GFP and mCherry-GBP were used in a 3:1 ratio. mCherry-GBP-PP1γ expression plasmid was generated by subcloning 7–300 of PP1γ into an mCherry-GBP expression plasmid. Astrin mutants are described in^35^. Astrin fragments (694–1193 a.a and 851–1193 a.a) encoding vectors were constructed through Gibson assembly using Astrin full-length cDNA (Q96Q06). Plasmid vector sequences were confirmed by DNA sequencing. Both plasmid DNA and plasmid maps are deposited in Ximbio.com.

### Immunofluorescence studies

Cells were cultured on ø13 mm round coverslips (VWR; 631-0150). Unless specified, cells were fixed with ice-cold methanol for a minute. Following fixation, two quick washes with a wash buffer (1X PBS + 0.1% Tween 20) were performed, followed by three washes of 5 min each. Coverslips were incubated with (1X PBS + 0.1% Tween 20 + 1% BSA) for 20 min, before staining with primary antibodies overnight at 4 °C. For the study of Bub1pSpT and Knl1 MELpT at kinetochores of monopolar spindles, cells were fixed with Methanol, and Protease/Phosphatase Inhibitor Cocktail (Cell Signalling Technology; 5872 S; 1:100) was added to the blocking buffer. For other studies assessing phospho-epitopes, cells were treated with prewarmed PHEM buffers described previously^35^. Cells were stained with antibodies against α-Tubulin (Abcam; ab6160; 1:800 or 1:500), Cyclin B1 (Abcam; ab72; 1:1000), GFP (Roche; 1181446001; 1:800), mCherry (Thermo Scientific; M11217; 1:2000), SKAP (Atlas; HPA042027; 1:1000), Astrin (Novus; NB100-74638; 1:1000), GFP (Abcam; ab290; 1:1000), mCherry (Abcam; ab167453; 1:2000), CENP-T pS47 (Sigma; abe1846; 1:1000), Hec1 pS55 (Fisher; PA5-85846; 1:500), KNL1 MELpT (Kops Lab; 1:1000), Bub1 pSpT (Nilsson Lab; 1:1000), Bub1 (Abcam; Ab195268; 1:500), CASC5 (NOVUS; NB100-2586; 1:500)and CREST anti-sera (Europa; FZ90C-CS1058; 1:2000) were used. DAPI (Sigma) was used to co-stain DNA. All antibody dilutions were prepared using the blocking buffer.

### Deconvolution microscopy and image analysis

For all high-resolution live-cell imaging assays, cells were either transfected with plasmid vectors 24 h before imaging or directly transferred to imaging in Leibovitz’s L15 medium (Invitrogen; 11415064) with 10% FCS and antibiotics (Penicillin and Streptomycin). For the bipolar spindle and monopolar spindle cell studies, the media was supplemented with MG132 and incubated for 1 h or STLC for 4~5 h at 37 °C before imaging. For the live-cell anaphase study, 24 h after the plasmid transfection, cells were arrested with STLC for 5 h. STLC was washed out with pre-warmed Leibovitz’s L15 medium, and metaphase cells coexpressing YFP-Astrin and Nuf2-CFP were marked under the microscope. Multi-Z-plane live-cell images of anaphase were taken when sister kinetochores started to separate. For high-resolution live-imaging at least 6 *Z*-planes, 0.3 μm apart, were acquired using a 100X NA 1.40 oil immersion objective on an Applied Precision DeltaVision Core microscope equipped with a Cascade2 camera under EM mode. Imaging was performed at 37 °C using a full-stage incubation chamber set up to facilitate long-term imaging of normal G1-S transition and mitosis progression^4^, including spindle movements^82^ and microtubule dynamics^83^. For HeLa cells transiently expressing mKate2-Astrin and Nuf2-GFP, kinetochores were identified using the Nuf2-GFP signal. For HeLa cells transiently expressing YFP-Astrin and Nuf2-CFP, kinetochores were identified by Nuf2-CFP signal. Images of immunostained cells were acquired using 100X NA 1.4 objective on a DeltaVision Core microscope equipped with CoolSnap HQ Camera (Photometrics^TM^). Volume rendering was performed for 3D analysis of kinetochore-microtubule attachment status (as in Ref. ^27^). Deconvolution and volume rendering of images were performed using SoftWorx^TM^ (Version 6.5.2).

The Fluorescence Recovery After Photobleaching (FRAP) experiments were performed on the Deltavision Core microscope using Quantifiable laser module components (488 nm laser). Target points were identified and then bleached with a pulse duration of 1 s and a laser power of 20% as in Ref. ^84^. Soon after bleaching, time-lapse imaging was initiated with an image acquired every 6 (Fig. 2a) or 15 (Fig. 2c) minutes through three Z-planes (0.9 µm), using a 100 x  NA objective with a Cascade2 camera under EM mode.

Images were analysed using FIJI^TM^ Software (NCBI, Version 2.0.0-rc-69/1.52p) using a 6 × 6 pixel circle area. Ratios were calculated using Excel^TM^ (Microsoft) and graphs plotted using Prism6^TM^ (GraphPad).

### Super-resolution microscopy and image analysis

For super-resolution microscopy, fixed cell images were acquired using a Structured Illumination microscope, ZEISS Elyra 7 with Lattice SIM² module. For each cell, at least 34 Z-planes (102 images in total spanning ~12.7 µm) were taken. SIM^2^ 3D image processing with the low SNR was performed and then images were analysed using ZEN 3.3 (blue edition) software. To measure the distance between YFP-Astrin and CREST, a straight line passing through the centres of both YFP and CREST signals was drawn using the profile function of the ZEN 3.3 (blue edition) software. Kinetochores displaying YFP-Astrin and CREST with their maximal cross-sectional areas in the same Z-plane were considered to allow lateral X-Y distance measurement between the YFP and CREST signal intensity peak. Representative images were cropped using FIJI Software (NCBI).

### Statistical analysis

Data were plotted using Prism-8 (GraphPad) software. Statistical analysis was performed using Prism-8 (GraphPad) using data from independent biological replicates or repeats as indicated. ‘*’ and ‘ns’ indicate statistically significant and insignificant differences in the tests, respectively. *P* ≥ 0.05 (ns), *P* < 0.05 (*), *P* < 0.01 (**), *P* < 0.001 (***) and *P* < 0.0001 (****).

### Reporting Summary

Further information on research design is available in the Nature Research Reporting Summary linked to this article.

## Supplementary information

Supplementary information

Reporting Summary

## Data Availability

Quantitative metrics and associated raw data generated in this study are available as a Source Data file provided with this paper. Raw image datasets generated are available freely for non-commercial research purposes from the corresponding author upon reasonable request. Source data are provided with this paper.

## Source data

Source Data
