# Supplementary material for: Counteraction between Astrin-PP1 and Cyclin-B-CDK1 pathways protects chromosome-microtubule attachments independent of biorientation
Source: Nat Commun. 2021 Dec 1;12:7010. doi: 10.1038/s41467-021-27131-9 (PMC8636589; doi:10.1038/s41467-021-27131-9)
Supplement: Supplementary file 1 — Supplementary information [file 41467_2021_27131_MOESM1_ESM.pdf]

Supplementary Information

Counteraction between Astrin-PP1 and Cyclin-B-CDK1 pathways protects chromosome-microtubule attachments independent of biorientation

Xinhong Song*, Duccio Conti*, Roshan L. Shrestha, Dominique Braun and Viji M. Draviam^

^Correspondence to v.draviam@qmul.ac.uk

*Equal contribution

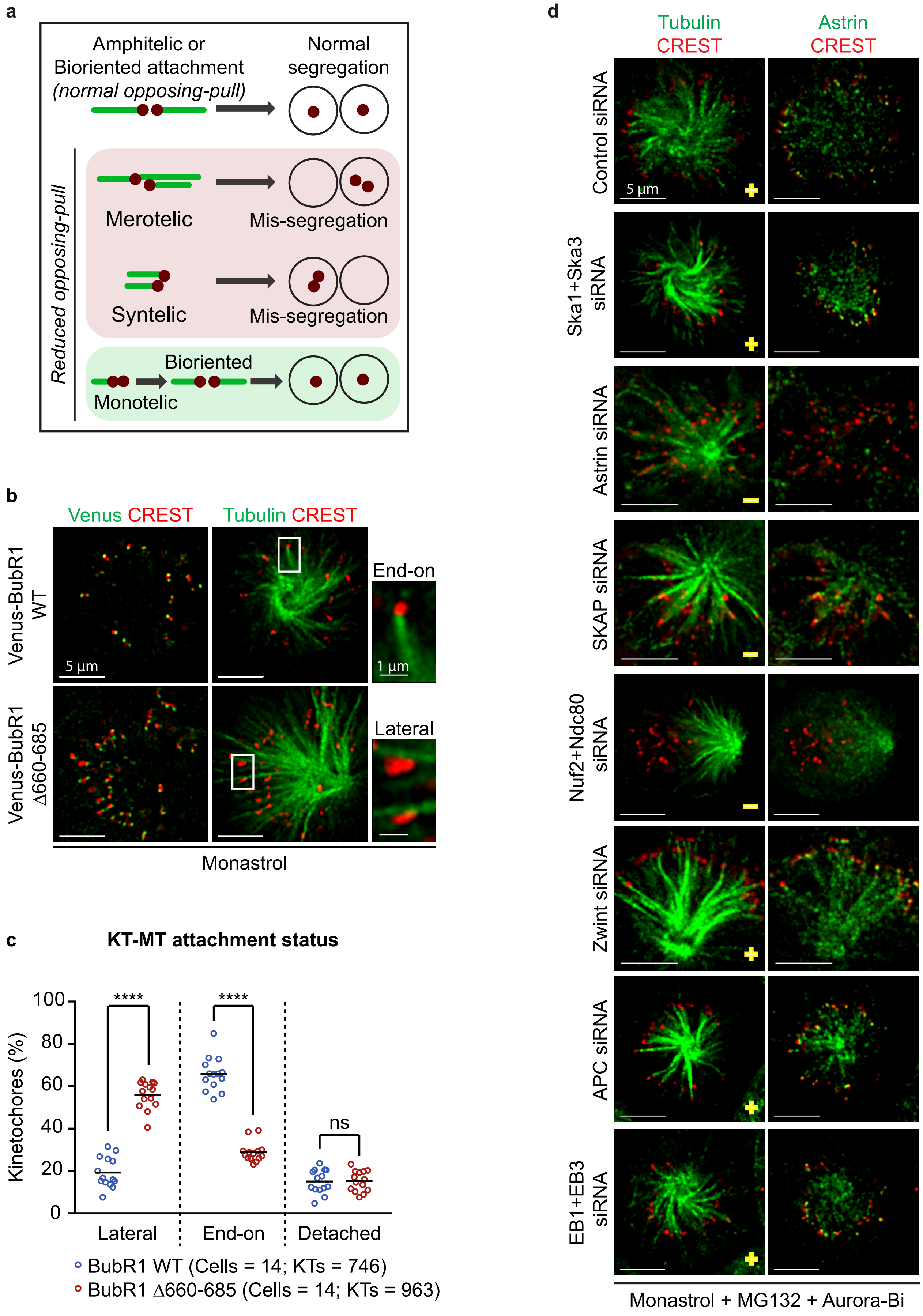

a
Amphitelic or
Bioriented attachment
(normal opposing-pull)
Normal
segregation
Reduced opposing-pull
Merotelic
Mis-segregation
Syntelic
Mis-segregation
Bioriented
Monotelic
b
Venus CREST
Tubulin CREST
Venus-BubR1 WT
Venus-BubR1 Δ660-685
5 µm
End-on
1 µm
Lateral
Monastrol
c
KT-MT attachment status
Kinetochores (%)
100
80
60
40
20
0
****
****
ns
Lateral
End-on
Detached
BubR1 WT (Cells = 14; KTs = 746)
BubR1 Δ660-685 (Cells = 14; KTs = 963)
d
Tubulin CREST
Astrin CREST
Control siRNA
5 µm
Ska1+Ska3 siRNA
Astrin siRNA
SKAP siRNA
Nuf2+Ndc80 siRNA
Zwint siRNA
APC siRNA
EB1+EB3 siRNA
Monastrol + MG132 + Aurora-Bi

Supplementary Figure 1: Regulators of non-bioriented end-on attachments

a, Cartoon of non-bioriented attachments that can cause Chromosomal Instability (CIN). Syntelic and Merotelic attachments must be resolved (box in red) while Monotelic attachments should be transiently protected (box in green) to allow normal biorientation. Reduced opposing-pull triggers the correction of attachment errors by Aurora-B; how monotelic attachments lacking opposing-pull are spared is unclear. **b,** Images showing kinetochore-microtubule attachment status in monopolar spindles of cells depleted of BubR1 and expressing either Venus-tagged BubR1-WT or a BubR1 mutant that can not deliver the B56 phosphatase. Cells were Monastrol treated for 3 hours prior to immunostaining with antibodies against Tubulin, GFP or CREST anti-sera. **c,** Scatter plot of percentage of end-on attached kinetochores in cells treated as in **b**, shows a reduction in the percentage of end-on attachments in cells lacking BubR1-B56 phosphatase at the kinetochore. Each dot represents a value from one cell. Horizontal black bars indicate mean values across three independent repeats. "*" and "ns" indicate significant and insignificant statistical differences, respectively determined using nonparametric Mann Whitney test. The exact p-values can be found in the Source data. **d,** RNAi-based screen for kinetochore-microtubule bridges required for stable non-bioriented attachments in the absence of Aurora-B activity. Cells were treated with the siRNA as indicated and exposed to Monastrol for 1 hour prior to a 15 minute exposure to Aurora-B and proteasome inhibitors (ZM447439 and MG132, respectively). Cells were fixed and immunostaining with antibodies against Tubulin, Astrin (end-on kinetochore marker) and CREST anti-sera. Images labelled '+' and '-' indicate the presence and absence of bouquet-like kinetochore arrangement at the ends of microtubules of monopolar spindles. For sample size, see Supplementary Table-1.

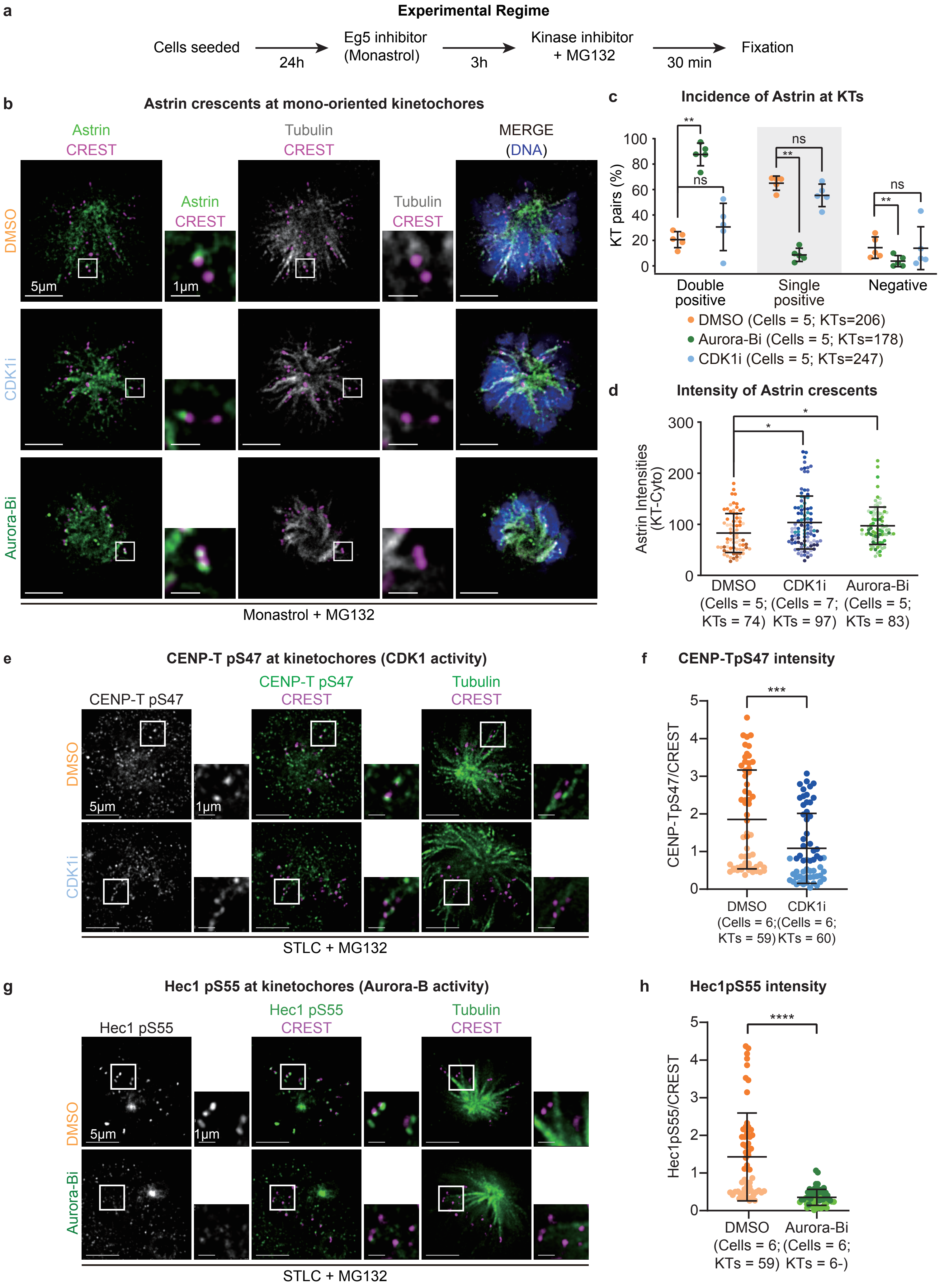
a
Experimental Regime
Cells seeded
24h
Eg5 inhibitor (Monastrol)
3h
Kinase inhibitor + MG132
30 min
Fixation
b
Astrin crescents at mono-oriented kinetochores
Astrin CREST
Tubulin CREST
MERGE (DNA)
DMSO
CDK1i
Aurora-Bi
5μm
1μm
Monastrol + MG132
c
Incidence of Astrin at KTs
KT pairs (%)
Double positive
Single positive
Negative
DMSO (Cells = 5; KTs=206)
Aurora-Bi (Cells = 5; KTs=178)
CDK1i (Cells = 5; KTs=247)
d
Intensity of Astrin crescents
Astrin Intensities (KT-Cyto)
DMSO (Cells = 5; KTs = 74)
CDK1i (Cells = 7; KTs = 97)
Aurora-Bi (Cells = 5; KTs = 83)
e
CENP-T pS47 at kinetochores (CDK1 activity)
CENP-T pS47
CENP-T pS47 CREST
Tubulin CREST
STLC + MG132
f
CENP-TpS47 intensity
CENP-TpS47/CREST
DMSO (Cells = 6; KTs = 59)
CDK1i (Cells = 6; KTs = 60)
g
Hec1 pS55 at kinetochores (Aurora-B activity)
Hec1 pS55
Hec1 pS55 CREST
Tubulin CREST
STLC + MG132
h
Hec1pS55 intensity
Hec1pS55/CREST
DMSO (Cells = 6; KTs = 59)
Aurora-Bi (Cells = 6; KTs = 6-)

Supplementary Figure 2: Unlike Aurora-B inhibition, CDK1 inhibition increases Astrin levels at kinetochore without disrupting error correction pathways.
a, Experimental regime: Cells were treated with inhibitors as indicated and immunostained with Astrin, Tubulin antibody and CREST anti-sera. **b,** Images of monopolar spindles show monooriented kinetochores in DMSO or CDK1i (Roscovitine) treated cells and syntelic kinetochores in Aurora-Bi (ZM447439) treated cells. **c,** Graph of percentage of kinetochore pairs showing syntelic (Astrin double positive), monotelic (Astrin single positive) and immature and unattached (Astrin negative) kinetochore pairs in cells treated as in **a**. Each dot represents a percentage value from one cell. **d,** Graph of intensity of Astrin at outer kinetochore in cells treated as in **a**. Colours in the graph **d** represent different cells. Each dot represents a value from one kinetochore. Black bars and whiskers in the subpanel **c** and **d** mark average value and standard deviation, respectively, across cells. '*' and 'ns' indicate statistically significant and insignificant differences from the Non-parametric Mann-Whitney test, respectively. **e** and **g**, Images of cells treated with STLC followed by MG132 and either CDK1 inhibitor (RO3306) or Aurora-B inhibitor (ZM447439) as indicated in **a** and immunostained with antibodies against Tubulin and either CENP-T pS47 (**e**) or Hec1pS55 (**g**) and CREST anti-sera. Scale bar as indicated. **f and h**, Graphs show the intensity changes in CENP-T pS47 (**f**) or Hec1 pS55 (**h**) levels at kinetochores following CDK1 or Aurora-B inhibition, respectively, determined using images as shown in **e** and **g**. Black bars and whiskers in the graphs mark average value and standard deviation, respectively, across two independent experimental repeats. Each dot represents a value from one kinetochore. '*' and 'ns' in the graphs **c**, **d**, **f** and **h** indicate statistically significant and insignificant differences, respectively, as determined using two-tailed Mann Whitney test. The exact p-values can be found in Source data. Colours in super-plot **f** and **h** represent independent experimental repeats.

a **Maintenance of end-on attachment**

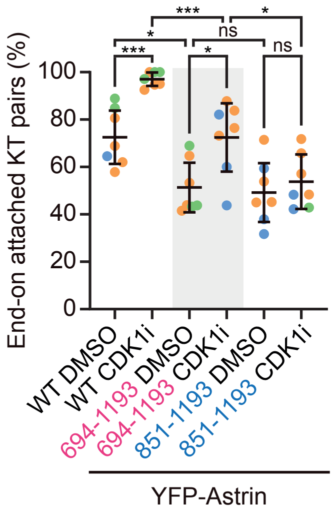

b **Sensing of end-on KTs**

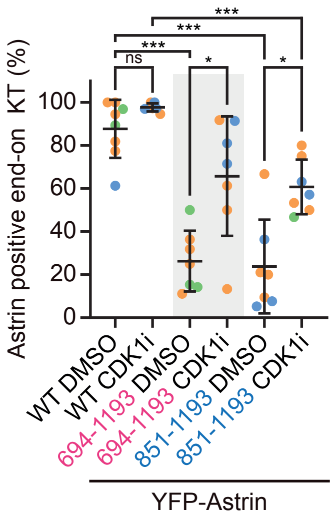

Supplementary Figure 3: Recruitment of Astrin C-terminal fragments at end-on kinetochores is negatively regulated by the CDK1 pathway

a, Scatter plot showing the proportion of end-on attached kinetochores assessed using immunostained images of cells treated as in Figure 3a. **b,** Scatter plot to ascertain the extent to which Astrin WT or fragments are recruited at end-on kinetochores (as a measure of ability to sense attachment status) in mono-oriented kinetochores of cells exposed to CDK1i (RO3306) or DMSO as indicated in Figure **3a**. Black bars and whiskers mark average value and standard deviation, respectively, across independent experimental repeats (n = 3). Each dot represents a value from one cell. '*' and 'ns' indicate statistically significant and insignificant differences, respectively, as determined using a two-tailed Mann Whitney test. Exact p-values can be found in source data.

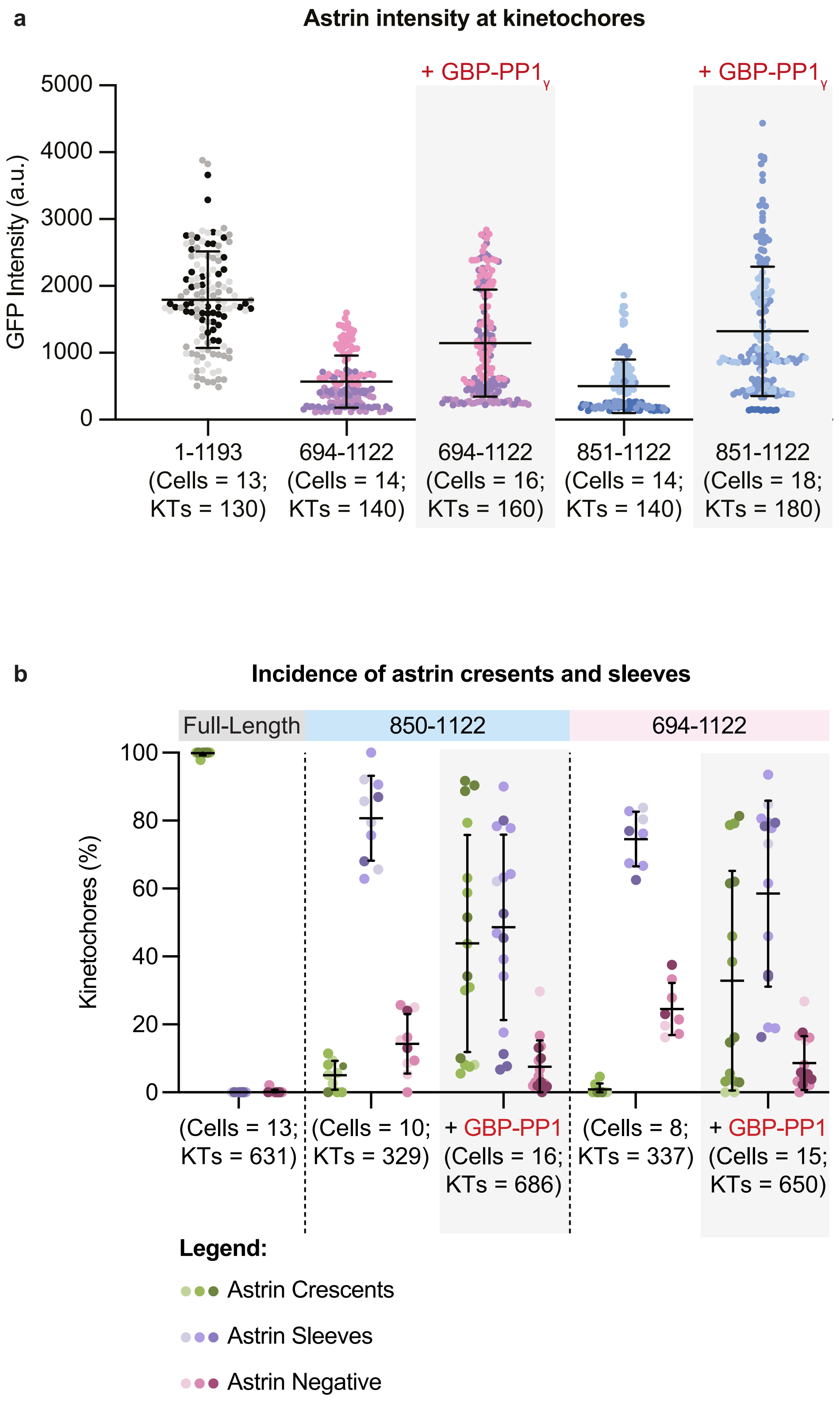
a
Astrin intensity at kinetochores
GFP Intensity (a.u.)
5000
4000
3000
2000
1000
0
+ GBP-PP1γ
+ GBP-PP1γ
1-1193 (Cells = 13; KTs = 130)
694-1122 (Cells = 14; KTs = 140)
694-1122 (Cells = 16; KTs = 160)
851-1122 (Cells = 14; KTs = 140)
851-1122 (Cells = 18; KTs = 180)
b
Incidence of astrin cresents and sleeves
Full-Length
850-1122
694-1122
Kinetochores (%)
100
80
60
40
20
0
(Cells = 13; KTs = 631)
(Cells = 10; KTs = 329)
+ GBP-PP1 (Cells = 16; KTs = 686)
(Cells = 8; KTs = 337)
+ GBP-PP1 (Cells = 15; KTs = 650)
Legend:
Astrin Crescents
Astrin Sleeves
Astrin Negative

Supplementary Figure 4: Microtubule-binding region of Astrin is important for Astrin's full enrichment at the outer kinetochore

a, Scatter plot of GFP intensities shows the recruitment extent of GFP-C-terminal tagged fragments of Astrin, as indicated in cells with or without GBP-PP1 coexpression (in red box). Each dot represents a value from one kinetochore. Different colours indicate values from 3 different data sets. Bars and whiskers indicate mean and standard deviation, respectively. **b,** Scatter plot of Astrin recruitment status showing the proportion of Astrin Crescent or Sleeve or no Astrin signal (negative) bearing kinetochores in cells expressing YFP-Astrin WT or deletion mutants with or without mCherry-GBP-PP1 co-expression as indicated. Black bars and whiskers mark average value and standard deviation, respectively, across three independent experimental repeats. Each dot represents a value from one cell. Colours of dots in **b** represent experimental repeats.

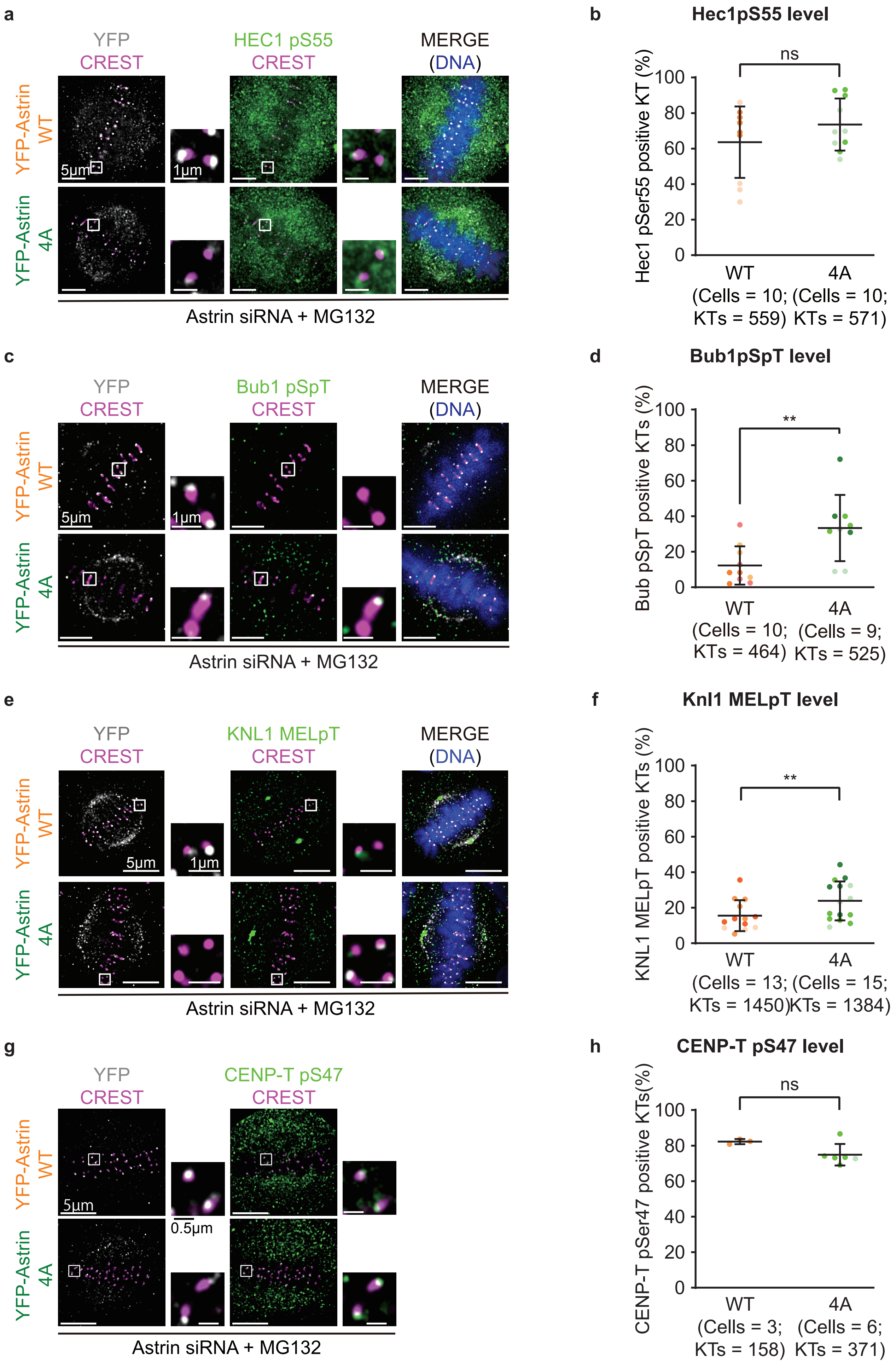
a
YFP
CREST
HEC1 pS55
CREST
MERGE
(DNA)
YFP-Astrin WT
YFP-Astrin 4A
5μm
1μm
Astrin siRNA + MG132
b
Hec1pS55 level
Hec1 pSer55 positive KT (%)
ns
100
80
60
40
20
0
WT
(Cells = 10; KTs = 559)
4A
(Cells = 10; KTs = 571)
c
YFP
CREST
Bub1 pSpT
CREST
MERGE
(DNA)
YFP-Astrin WT
YFP-Astrin 4A
5μm
1μm
Astrin siRNA + MG132
d
Bub1pSpT level
Bub pSpT positive KTs (%)
**
100
80
60
40
20
0
WT
(Cells = 10; KTs = 464)
4A
(Cells = 9; KTs = 525)
e
YFP
CREST
KNL1 MELpT
CREST
MERGE
(DNA)
YFP-Astrin WT
YFP-Astrin 4A
5μm
1μm
Astrin siRNA + MG132
f
Knl1 MELpT level
KNL1 MELpT positive KTs (%)
**
100
80
60
40
20
0
WT
(Cells = 13; KTs = 1450)
4A
(Cells = 15; KTs = 1384)
g
YFP
CREST
CENP-T pS47
CREST
YFP-Astrin WT
YFP-Astrin 4A
5μm
0.5μm
Astrin siRNA + MG132
h
CENP-T pS47 level
CENP-T pSer47 positive KTs(%)
ns
100
80
60
40
20
0
WT
(Cells = 3; KTs = 158)
4A
(Cells = 6; KTs = 371)

Supplementary Figure 5: Analysis of downstream substrates of Astrin-PP1
a, c, e and **g:** Images show the extent of phosphorylated Hec1 pS55 (**a**); Bub1 pSpT; (**c**); KNL1 MELpT(**e**) or CENP-T pS47 (**g**) at kinetochores of cells depleted of endogenous Astrin and expressing either Astrin-WT or Astrin-4A mutant as indicated. Cells were treated with MG132 for an hour prior to fixation for immunostaining with antibodies against GFP and phospho-epitopes as indicated and CREST anti-sera (a centromere marker). Scale bar as indicated. **b, d, f** and **h:** Graphs show the percentage of kinetochores positive for phosphorylated Hec1 pS55 (**b**); Bub1 pSpT(**d**); KNL1 MELpT (**f**) or CENP-T pS47 (**h**) as determined using images as shown in **a**, **c**, **e** and **g**, respectively. Black bars and whiskers in the subpanel **b, d, f** and **h** mark average value and standard deviation, respectively, across independent experimental repeats (n = 3 repeats (Hec1 pS55, Bub1 pSpT and Knl1 MELpT) and 2 repeats (CENP-T pS47). Each dot represents a value from one cell. ‘*' and ‘ns’ indicate statistically significant and insignificant differences, respectively, as determined using a two-sided Mann Whitney test. Colours in super-plot represent independent experimental repeats.

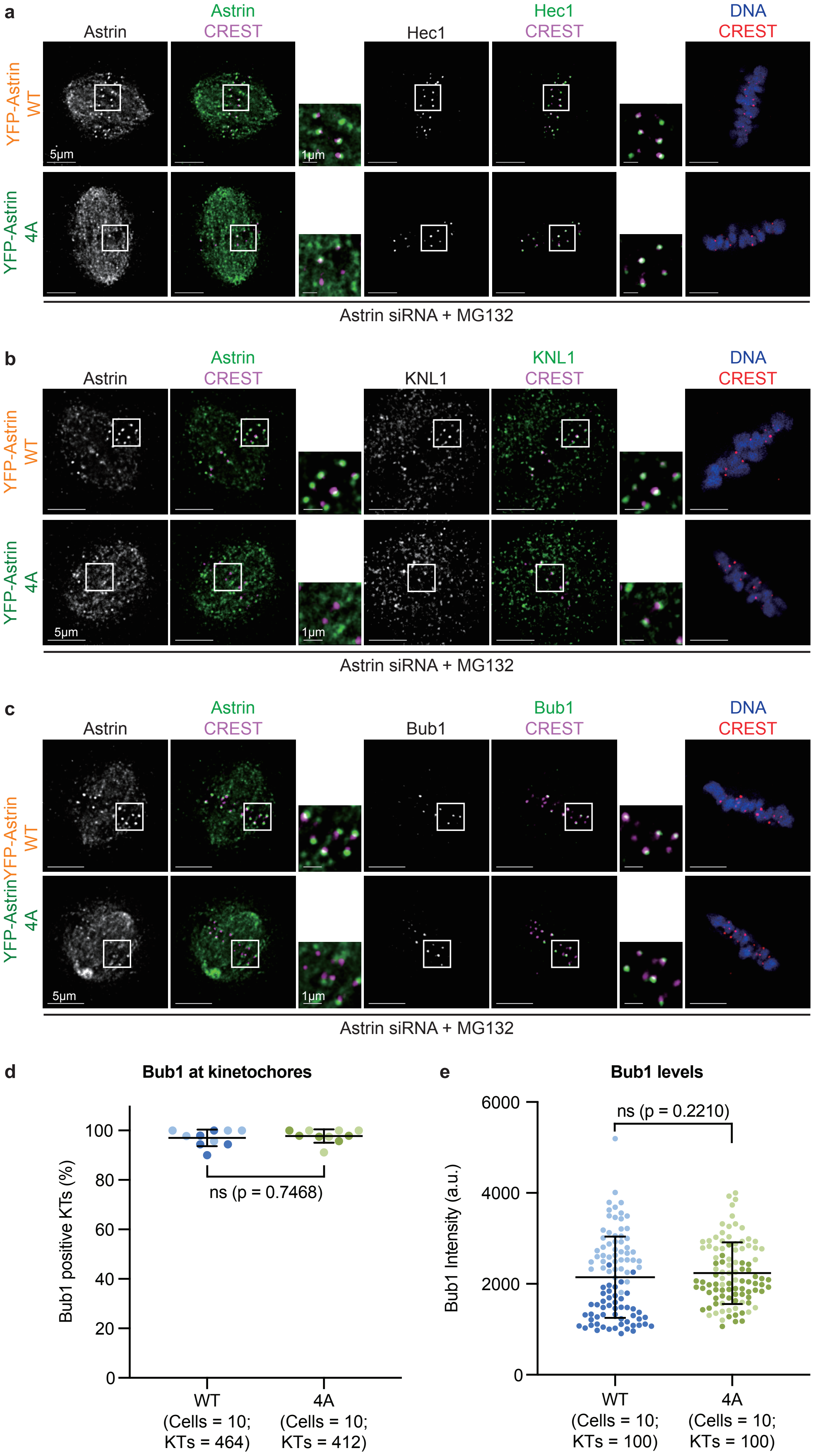
a
Astrin
Astrin
CREST
Hec1
Hec1
CREST
DNA
CREST
YFP-Astrin WT
YFP-Astrin 4A
5µm
1µm
Astrin siRNA + MG132
b
Astrin
Astrin
CREST
KNL1
KNL1
CREST
DNA
CREST
YFP-Astrin WT
YFP-Astrin 4A
5µm
1µm
Astrin siRNA + MG132
c
Astrin
Astrin
CREST
Bub1
Bub1
CREST
DNA
CREST
YFP-Astrin WT
YFP-Astrin 4A
5µm
1µm
Astrin siRNA + MG132
d
Bub1 at kinetochores
Bub1 positive KTs (%)
100
80
60
40
20
0
ns (p = 0.7468)
WT
(Cells = 10;
KTs = 464)
4A
(Cells = 10;
KTs = 412)
e
Bub1 levels
Bub1 Intensity (a.u.)
6000
4000
2000
0
ns (p = 0.2210)
WT
(Cells = 10;
KTs = 100)
4A
(Cells = 10;
KTs = 100)

Supplementary Figure 6: Outer-kinetochore proteins Hec1, KNL1 and Bub1 in Astrin-4A mutant expressing cells

a - c: Images show the presence of Hec1 (**a**), KNL1 (**b**) or Bub1 (**c**) at kinetochores of cells depleted of endogenous Astrin and expressing either YFP-Astrin WT or 4A mutant as indicated. Cells were treated with MG132 for an hour prior to fixation for immunostaining with antibodies as indicated and CREST anti-sera (a centromere marker) and DAPI to stain DNA. Scale bar as indicated. **d,** Graph showing the proportion of kinetochores positive for Bub1 at kinetochores. **e,** Graph shows the intensities of Bub1 signals in kinetochores of cells treated as in **c**. Black bars and whiskers in the subpanel **d** and **e** mark average value and standard deviation, respectively, across two independent experimental repeats. Each dot represents a value from one cell. '*' and 'ns' indicate statistically significant and insignificant differences, respectively as determined using the two sided Mann Whitney test. Colours in super-plot represent independent experimental repeats.

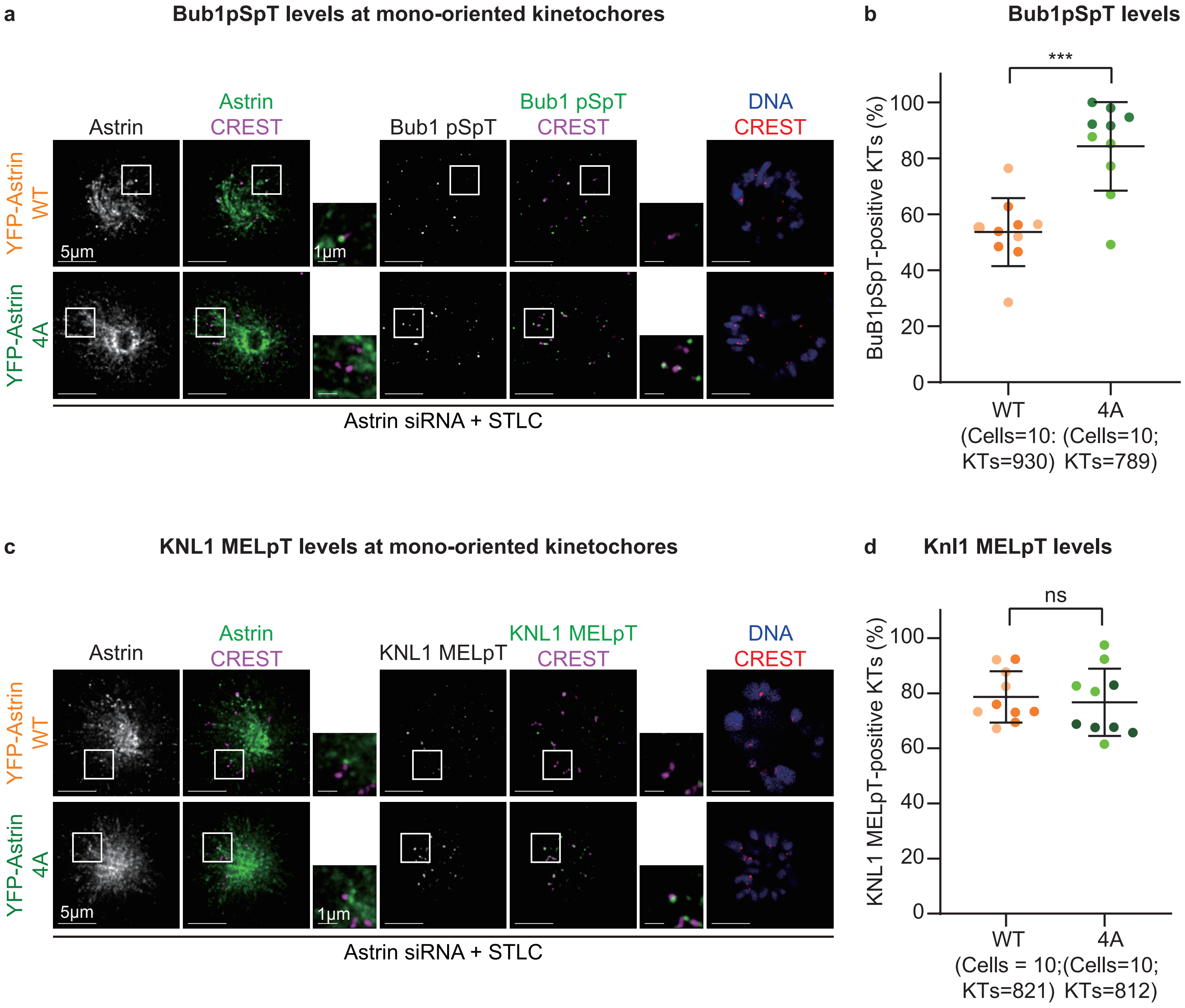

a
Bub1pSpT levels at mono-oriented kinetochores
Astrin
Astrin
CREST
Bub1 pSpT
Bub1 pSpT
CREST
DNA
CREST
YFP-Astrin WT
YFP-Astrin 4A
5µm
1µm
Astrin siRNA + STLC
b
Bub1pSpT levels
***
BuB1pSpT-positive KTs (%)
0
20
40
60
80
100
WT
(Cells=10: KTs=930)
4A
(Cells=10; KTs=789)
c
KNL1 MELpT levels at mono-oriented kinetochores
Astrin
Astrin
CREST
KNL1 MELpT
KNL1 MELpT
CREST
DNA
CREST
YFP-Astrin WT
YFP-Astrin 4A
5µm
1µm
Astrin siRNA + STLC
d
Knl1 MELpT levels
ns
KNL1 MELpT-positive KTs (%)
0
20
40
60
80
100
WT
(Cells = 10; KTs=821)
4A
(Cells=10; KTs=812)

Supplementary Figure 7: Analysis of phospho-changes downstream of Astrin-PP1 in monoriented kinetochores

a and **c:** Images show the extent of phosphorylated Bub1 pSpT (**a**) or KNL1 MELpT (**c**) at kinetochores of cells depleted of endogenous Astrin and expressing either YFP-Astrin WT or YFP-Astrin 4A mutant as indicated. Cells were treated with STLC for 5 hours prior to fixation for immunostaining with antibodies against GFP and phospho-epitopes as indicated and CREST anti-sera (a centromere marker) and DAPI to stain DNA. Scale bar as indicated. **b** and **d,** Graphs show the percentage of kinetochores positive for phosphorylated Bub1 pSpT(b) or KNL1 MELpT (d) as determined using images as shown in **a** and **c**, respectively. Centre of black bars and whiskers in the subpanel **b** and **d** mark average value and standard deviation, respectively, across two independent experimental repeats. Each dot represents a value from one cell. '*' and 'ns' indicate statistically significant and insignificant differences, respectively, determined using a two-sided Mann Whitney test . Colours in the super-plot b and d represent independent experimental repeats.

Supplementary Table 1

Candidate name	KT-MT attachment (MT-end tethered)				Bouquet arrangement	Astrin or SKAP at KT				Multinucleated cells				siRNA sequence 1	siRNA sequence 2
	I	II	III	IV		I	II	III	IV	I	II	III	IV		
Negative	Pos	Pos	Pos	Pos	Pos	Pos	Pos	Pos	Pos	13%	5%	0%	0%		
Astrin	Neg	Neg	Neg	Neg	Neg	Neg	Neg	Neg	Neg	9%	7%	16%	2%	TCCCGACAACTCACAGAGAAA	
SKAP	Neg	Neg	Neg	Neg	Neg	Less	Less	Less	Less	30%	44%	7%	10%	GAAAGAGTCCGATTCCTAG	
CENP-T	Pos	Pos	Pos	Pos	Pos	Pos	Pos	Pos	Pos	59%	64%	39%	44%	CAAGAGAGCAGTTGCGGCA	
Mis12	Pos	Pos	Pos	Pos	Pos	Pos	Pos	Pos	Pos	11%	3%	0%	9%	Life Technologies HSS128129	
Ndc80+Nuf2	Neg	Neg	Neg	Neg	Neg	Neg	Neg	Neg	Neg	36%	66%	57%	50%	AAACACTTACTGCTCTCTCCAGT	GAGAAAUACCACGACGGUAUUGAAA
Zwint	Pos	Pos	Pos	Pos	Pos	Pos	Pos	Pos	Pos	19%	29%	38%	30%	AAGCTGCTCTGCAGCCAGCTT	
Ska1	Pos	Pos	Pos	Pos	Pos	Pos	Pos	Pos	Pos	0%	23%	23%	8%	CCCGCTTAACCTAUAATCAAA	
Ska3	Pos	Pos	Pos	Pos	Pos	Pos	Pos	Pos	Pos	0%	0%	5%	6%	AGACAAACATGAACATTAA	
EB1+EB3	Pos	Pos	Pos	Pos	Pos	Pos	Pos	Pos	Pos	11%	13%	22%	17%	TCTGACAAAGATCGAACAG	ACTATGATGGAAAGGATTAC
APC	Pos	Pos	Pos	Pos	Pos	Pos	Pos	Pos	Pos	16%	22%	26%	14%	GTGGCAGGAAGCTCATGAA	
chTOG1	Pos	Pos	Pos		Neg	Pos	Pos	Pos		*	*	*		AAGAGCAGUCGCAAAUGAAGC	
CLIP170	Pos	Pos	Pos		Pos	Pos	Pos	Pos		*	*	*		AAGCUAAUGGCCUGCAGACAA	
CENP-E	Pos	Pos	Pos		Pos	Pos	Pos	Pos		*	*	*		AAACACUUACUGCUCUCUCCAGUUU	

Supplementary Table 1: RNAi screen to identify KT-MT bridging proteins needed for maintaining end-on attachments despite the absence of Aurora-B activity. List indicates kinetochore proteins, siRNA oligo sequences used for RNAi, multinucleation status of cells (DAPI (nuclear) and Tubulin (cytoplasmic) staining), bouquet arrangement of kinetochores (indicative of end-tethered kinetochores) in cells immunostained using antibody against tubulin and Astrin and CREST anti-sera following siRNA treatment. '*' indicates cells predominantly arrested in mitosis with uncongressed chromosomes. For multinucleate phenotype, a total of 100 interphase cells were counted in every experimental repeat. For kinetochore-microtubule attachment (bouquet arrangement) and Astrin or SKAP at kinetochore phenotypes, a total of at least 10 monopolar spindles were assessed in every experimental repeat. 'Pos' (positive) refers to cases where at least 40% of assessed mitotic cells displayed a phenotype; the rest were characterised as 'Neg' (negative). I to IV represents independent experimental repeats.
