# Supplementary material for: Counteraction between Astrin-PP1 and Cyclin-B-CDK1 pathways protects chromosome-microtubule attachments independent of biorientation
Source: Nat Commun. 2021 Dec 1;12:7010. doi: 10.1038/s41467-021-27131-9 (PMC8636589; doi:10.1038/s41467-021-27131-9)
Supplement: Supplementary file 2 — Reporting Summary [file 41467_2021_27131_MOESM2_ESM.pdf]

nature research

Corresponding author(s): Viji M. Draviam

Last updated by author(s): 18 Oct 2021

Reporting Summary

Nature Research wishes to improve the reproducibility of the work that we publish. This form provides structure for consistency and transparency in reporting. For further information on Nature Research policies, see our Editorial Policies and the Editorial Policy Checklist.

Statistics

For all statistical analyses, confirm that the following items are present in the figure legend, table legend, main text, or Methods section.

n/a	Confirmed	
☐	☒	The exact sample size (*n*) for each experimental group/condition, given as a discrete number and unit of measurement
☐	☒	A statement on whether measurements were taken from distinct samples or whether the same sample was measured repeatedly
☐	☒	The statistical test(s) used AND whether they are one- or two-sided<br>*Only common tests should be described solely by name; describe more complex techniques in the Methods section.*
☒	☐	A description of all covariates tested
☒	☐	A description of any assumptions or corrections, such as tests of normality and adjustment for multiple comparisons
☐	☒	A full description of the statistical parameters including central tendency (e.g. means) or other basic estimates (e.g. regression coefficient) AND variation (e.g. standard deviation) or associated estimates of uncertainty (e.g. confidence intervals)
☐	☒	For null hypothesis testing, the test statistic (e.g. F, t, r) with confidence intervals, effect sizes, degrees of freedom and P value noted<br>*Give P values as exact values whenever suitable.*
☒	☐	For Bayesian analysis, information on the choice of priors and Markov chain Monte Carlo settings
☒	☐	For hierarchical and complex designs, identification of the appropriate level for tests and full reporting of outcomes
☒	☐	Estimates of effect sizes (e.g. Cohen's *d*, Pearson's *r*), indicating how they were calculated

Our web collection on statistics for biologists contains articles on many of the points above.

Software and code

Policy information about availability of computer code

Data collection	SoftWorx (DV core) was used for the collection of microscopy images.
Data analysis	Image analysis was performed using either SoftWorx or ImageJ softwares.

For manuscripts utilizing custom algorithms or software that are central to the research but not yet described in published literature, software must be made available to editors and reviewers. We strongly encourage code deposition in a community repository (e.g. GitHub). See the Nature Research guidelines for submitting code & software for further information.

Data

Policy information about availability of data

All manuscripts must include a data availability statement. This statement should provide the following information, where applicable:
- Accession codes, unique identifiers, or web links for publicly available datasets
- A list of figures that have associated raw data
- A description of any restrictions on data availability

Quantitative analysis and associated metrics are available as source data. Raw image datasets generated are available freely for non-commercial research purposes from the corresponding author upon reasonable request

Field-specific reporting

Life sciences study design

All studies must disclose on these points even when the disclosure is negative.

Sample size	Sample size was not statistically determined. For single-cell studies, at least 40 kinetochores with high-quality signal were analysed as a good representation of the overall phenotype. This cut-off value was established as it allowed the analysis of at least 50% of human kinetochores in HeLa cells, providing a robust interpretation of Astrin localisation phenotype.
Data exclusions	For kinetochore-microtubule attachment analysis in high-resolution microscopy images, image quality of each cell was assessed based on the percentage of attachments that were scored as "unknown". Cells where more than 15% of kinetochores were scored as "unknown" where excluded from the final analysis as considered of poor quality. For the rest of the experiments, no data was excluded. Only experimental repeats that were unsuccessful due to change in protocol, technical problems, or lack of controls were disregarded.
Replication	Each experiment was repeated at least two or three times (as indicated in the figure legends and methods). Where appropriate, data from different repeats within the same sample was plotted using different colours to show experimental variability together with standard deviation error bars.
Randomization	Within each experimental regime, cells were seeded and randomly assigned to a siRNA regime and/or a drug regime. For siRNA experiments, control siRNA cells were assigned at the beginning of the experiments. For drugs regimes, the solvent control (DMSO) condition was assigned at the beginning of the treatment.
Blinding	Not applicable. Blinding is not applicable as several other phenotypes that are coincident with perturbation cannot be masked.

Reporting for specific materials, systems and methods

We require information from authors about some types of materials, experimental systems and methods used in many studies. Here, indicate whether each material, system or method listed is relevant to your study. If you are not sure if a list item applies to your research, read the appropriate section before selecting a response.

Materials & experimental systems

n/a	Involved in the study
☐	☒ Antibodies
☐	☒ Eukaryotic cell lines
☒	☐ Palaeontology and archaeology
☒	☐ Animals and other organisms
☒	☐ Human research participants
☒	☐ Clinical data
☒	☐ Dual use research of concern

Methods

n/a	Involved in the study
☒	☐ ChIP-seq
☒	☐ Flow cytometry
☒	☐ MRI-based neuroimaging

Antibodies

Antibodies used	a-Tubulin (Abeam; ab6160); Cyclin Bl (Abeam; ab72); GFP (Abeam; ab290); GFP (Roche; 1181446001); mCherry (Abeam; ab167453); mCherry (Thermo Scientific; M11217); SKAP (Atlas; HPA042027); Astrin (Novus; NBl00-74638); CENP-T pSer47 (Sigma; abe1846); Heel pSerSS (Fisher Scientific; PAS-85846); KNLl MELpT (donated by the Kops Lab, Nijenhuis et al., 2014); Bubl pSpT (donated by Nilsson Lab); CREST antisera (Europa; FZ90C-CS1058); KNLl (Novus; NBl00-2586); Bubl (Abeam; ab195268)
Validation	All antibodies were validated by the manufacturers using molecular weight markers in western blots and immunofluorescence images. KNLl MELpT antibody was generated and validated by the Kops lab in the following published study: Nijenhuis et al., 2014. Bubl pSpT antibody was generated and validated by the Nilsson lab in the following published study: Zhang et al., 2017.

Eukaryotic cell lines

Policy information about cell lines

Cell line source(s)	Both HeLa and RPEl parental cell lines were acquired from ATCC. HeLa Flip-In Venus-BubRl cell lines were donated by the Nilsson lab (published in Kruse et al., 2013).
Authentication	None of the cell lines were authenticated by the authors. He La Flip-In Venus-BubRl cell lines were authenticated by the Nilsson lab using fusion protein expression analysis.
Mycoplasma contamination	RPEl parental cell line and HeLa cell line was tested and found to be negative for Mycoplasma contamination 5 years ago.
Commonly misidentified lines (See ICLAC register)	No commonly misidentified lines were used in the study.
